# Pangolin Genomes Offer Key Insights and Resources for the World’s Most Trafficked Wild Mammals

**DOI:** 10.1093/molbev/msad190

**Published:** 2023-10-05

**Authors:** Sean P Heighton, Rémi Allio, Jérôme Murienne, Jordi Salmona, Hao Meng, Céline Scornavacca, Armanda D S Bastos, Flobert Njiokou, Darren W Pietersen, Marie-Ka Tilak, Shu-Jin Luo, Frédéric Delsuc, Philippe Gaubert

**Affiliations:** Laboratoire Evolution et Diversité Biologique (EDB)— IRD-UPS-CNRS, Université Toulouse III, Toulouse, France; Institut des Sciences de l'Évolution de Montpellier (ISEM), Université de Montpellier, CNRS, IRD, Montpellier, France; Laboratoire Evolution et Diversité Biologique (EDB)— IRD-UPS-CNRS, Université Toulouse III, Toulouse, France; Laboratoire Evolution et Diversité Biologique (EDB)— IRD-UPS-CNRS, Université Toulouse III, Toulouse, France; The State Key Laboratory of Protein and Plant Gene Research of Life Sciences, Peking-Tsinghua Center for Life Sciences, Peking University, Beijing, China; Institut des Sciences de l'Évolution de Montpellier (ISEM), Université de Montpellier, CNRS, IRD, Montpellier, France; Mammal Research Institute, Department of Zoology & Entomology, University of Pretoria, Pretoria, South Africa; Laboratoire de Parasitologie et Ecologie, Faculté des Sciences, Université de Yaoundé I, Yaoundé, Cameroon; Mammal Research Institute, Department of Zoology & Entomology, University of Pretoria, Pretoria, South Africa; Institut des Sciences de l'Évolution de Montpellier (ISEM), Université de Montpellier, CNRS, IRD, Montpellier, France; The State Key Laboratory of Protein and Plant Gene Research of Life Sciences, Peking-Tsinghua Center for Life Sciences, Peking University, Beijing, China; Institut des Sciences de l'Évolution de Montpellier (ISEM), Université de Montpellier, CNRS, IRD, Montpellier, France; Laboratoire Evolution et Diversité Biologique (EDB)— IRD-UPS-CNRS, Université Toulouse III, Toulouse, France; CIIMAR/CIMAR, Centro Interdisciplinar de Investigação Marinha e Ambiental, Universidade 16 do Porto, Terminal de Cruzeiros do Porto de Leixões, Porto, Portugal

**Keywords:** pholidota, full genomes, genomic diversity, conservation, ancient admixture, novel taxon

## Abstract

Pangolins form a group of scaly mammals that are trafficked at record numbers for their meat and purported medicinal properties. Despite their conservation concern, knowledge of their evolution is limited by a paucity of genomic data. We aim to produce exhaustive genomic resources that include 3,238 orthologous genes and whole-genome polymorphisms to assess the evolution of all eight extant pangolin species. Robust orthologous gene-based phylogenies recovered the monophyly of the three genera and highlighted the existence of an undescribed species closely related to Southeast Asian pangolins. Signatures of middle Miocene admixture between an extinct, possibly European, lineage and the ancestor of Southeast Asian pangolins, provide new insights into the early evolutionary history of the group. Demographic trajectories and genome-wide heterozygosity estimates revealed contrasts between continental versus island populations and species lineages, suggesting that conservation planning should consider intraspecific patterns. With the expected loss of genomic diversity from recent, extensive trafficking not yet realized in pangolins, we recommend that populations be genetically surveyed to anticipate any deleterious impact of the illegal trade. Finally, we produce a complete set of genomic resources that will be integral for future conservation management and forensic endeavors for pangolins, including tracing their illegal trade. These comprise the completion of whole-genomes for pangolins through the hybrid assembly of the first reference genome for the giant pangolin (*Smutsia gigantea*) and new draft genomes (∼43x–77x) for four additional species, as well as a database of orthologous genes with over 3.4 million polymorphic sites.

## Introduction

Genomics is being prioritized in wildlife research as it provides genome-wide data for more accurate inferences on species or population delimitation, demographic parameters, diversity, historical trajectories, and the adaptive capacity to global changes (Allendorf et al. 2010). Although transforming this research into conservation practice is yet to be common (Shafer et al. 2015), the gap is closing (Garner et al. 2016; Formenti et al. 2022; Paez et al. 2022).

Pangolins are a group of mammals in the order Pholidota harboring eight extant species (four each in Africa and Asia; Gaubert et al. 2020) that have become a taxon of great public interest and conservation concern in recent years (Pietersen and Challender 2020; Heighton and Gaubert 2021). This is mainly due to them being the most trafficked wild mammals on Earth (Heinrich et al. 2017) and a recent, incorrect, suggestion that they may have been linked to the COVID-19 pandemic (Frutos et al. 2020; Lam et al. 2020; Lee et al. 2020). Despite their dire conservation circumstances, pangolins are considered to be understudied with major gaps in basic species or population research (Pietersen and Challender 2020; Heighton and Gaubert 2021). Even with a long-standing interest in their taxonomy, particularly in light of their convergent evolution with South American anteaters (Xenarthra: Miyamoto and Goodman 1986; Wyss et al. 1987; Murphy et al. 2001; Delsuc et al. 2002), phylogenetic studies focusing on pangolins have been incomplete. They have either been restricted to single marker or mitochondrial genomes, limited by taxon coverage, or clouded by incorrect taxonomic sampling (Yu et al. 2011; Du Toit et al. 2014; Gaubert and Antunes 2015; Hassanin et al. 2015; du Toit et al. 2017). The most comprehensive phylogenetic study to date was inferred using a dataset of mitogenomes and nine nuclear genes encompassing all eight species (Gaubert et al. 2018).

As for genome-wide inferences for pangolins, the majority of studies undertaken have been non-NGS (next-generation sequencing) related and have used traditional methods like karyotyping (Ray-Chaudhuri et al. 1969; Su et al. 1994; Che et al. 2008; Zhihai et al. 2016). In fact, only two pangolin species (Chinese pangolin; *Manis pentadactyla* and Sunda pangolin; *M. javanica*) have published full genomes, both of which have become the subject of crucial conservation genomic studies (Choo et al. 2016; Nash et al. 2018; Hu, Hao et al. 2020). These studies have mainly focused on demographic history to determine the consequences of recent population fluctuations due to climatic oscillations (Hu, Hao et al. 2020; Wei et al. 2022). However, they have also delved into comparisons of diversity, inbreeding, mutational load amongst populations as well as population structuring for geographic assignment of illegally traded individuals (Hu, Hao et al. 2020; Wei et al. 2022). Despite the thoroughness of these studies on the two species, our understanding of the genome-wide evolution of pangolins is still taxonomically limited, which compromises the potential of utilizing genetic markers for conservation and management purposes of the entire order (Allendorf et al. 2010; Kotze et al. 2020).

Producing genome-wide inferences of pangolins is a challenging task. First, their elusive behavior and tropical distributions render genetic sampling to be time-consuming and costly. Second, the phylogenetic isolation of the group from other mammalian orders, the limited fossil records, and the deep divergence within pangolins (Gaudin et al. 2009; Gaubert et al. 2018), pose methodological hurdles. Despite Pholidota being sister to the order Carnivora, the two orders are estimated to have started to diverge from around 78.9–76 million years ago (Zhou et al. 2011; Gaubert et al. 2018), thus making it difficult to incorporate outgroup taxa for genomic inferences of divergence and introgression. Compounded by this is the deep divergence (± 37.9 million years ago) of Asian and African pangolins (Gaubert et al. 2018) which may introduce ascertainment bias to genomic inferences depending on which continental clade the reference genome is a part of (Günther and Nettelblad 2019; Bohling 2020). Therefore, alternative approaches to the simple variant-based phylogenomic analyses based on the alignment of all taxa to a single pangolin reference genome need to be considered (Kapli et al. 2020; Prasad et al. 2022).

The basis of conservation genomics relies upon generating genomic data such as reference genomes and datasets of markers (e.g., homologous/orthologous genes, single nucleotide polymorphisms [SNPs]) used to make species and population inferences for application in conservation management (Allendorf et al. 2010; Formenti et al. 2022; Paez et al. 2022). With pangolins requiring conservation action, compounded by limited genomic information for the group, a gap needs to be filled. We thus aim to conduct the first full genome analysis on all eight pangolin species, to determine evolutionary relationships and demographic trends, identify key genetic parameters for species management, and develop a set of markers for future conservation genetic/genomic efforts. Additionally, we provide a high-quality hybrid assembled reference genome of the giant pangolin (*Smutsia gigantea*), an elusive fossorial species found in western and central Africa, which promises to be a key reference for the *Smutsia* genus and the African species alike.

## Results and Discussion

We sequenced, assembled, and annotated the first reference genome for the genus *Smutsia* (giant pangolin; *S. gigantea,* ∼87x) using a combination of long-read Nanopore sequencing and short-read Illumina sequencing (hybrid assembly). We also sequenced and assembled short read Illumina draft genomes for the black-bellied (*Phataginus tetradactyla,* ∼43x), Temminck's (*Smutsia temminckii,* ∼44x), Indian (*Manis crassicaudata,* ∼53x), and Philippine (*Manis culionensis,* ∼77x) pangolins (supplementary table S1, Supplementary Material online). These new genomic data along with previously published reference genomes of the remaining three species, namely, the white-bellied (*Phataginus tricuspis*), Sunda (*M. javanica*), and Chinese (*M. pentadactyla*) pangolins (supplementary table S1, Supplementary Material online), provide the first complete set of genomes for Pholidota. We also included a recently published genome of a pangolin of uncertain origin and taxonomy, seized from south-western China (Cao et al. 2021). In an effort to reduce ascertainment bias from mapping deeply divergent Asian and African species to a single reference (Albrechtsen et al. 2010; Lachance and Tishkoff 2013; Prasad et al. 2022), species were mapped to a representative genome from their respective clade (supplementary fig. S1, Supplementary Material online). The mapped genomes then underwent haplotype (haploid) and IUPAC ambiguity codes (diploid) consensus assignment before using mtDNA haplotypes and 3,238 entire single-copy orthologous autosomal genes (introns and exons) for phylogenomic and divergence time inferences.

### A Potential New Species of Pangolin From Asia

**Fig. 1.**
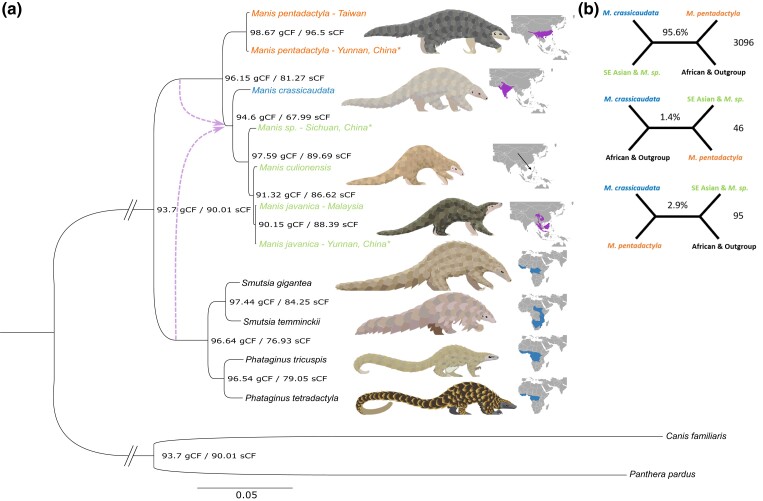
(*a*) Phylogenetic tree of extant pangolins based on the alignment(*s*) of 58,724,014 bp from 3,238 IUPAC consensus whole-gene markers. The phylogeny consists of 13 individuals from all eight species and is rooted with two representatives of the sister order Carnivora. The same topology was derived from a partitioned concatenated (supermatrix), nonpartitioned concatenated (supplementary fig. S2*a*, Supplementary Material online), and coalescent (summary tree; supplementary fig. S2*b*, Supplementary Material online) phylogenies, hence only the former is shown. All three phylogenies have full branch support for all nodes (100 for the concatenated phylogenies using 1,000 Felsenstein bootstrap replicates and one for the coalescent phylogeny using local posterior probability). Nodal numbers represent the concordance factors of genes (gCF) and sites (sCF off 100 quartets), which indicate the proportion of genes and sites that fit the current topology. Dotted lines with arrow heads indicate a suggested reticulation event based on maximum pseudolikelihood phylonetworks testing (supplementary fig. S5, Supplementary Material online). (*b*) The main species tree quartet and the two alternative gene tree quartet topologies (from top to bottom on the top right) were identified from the coalescent analysis (supplementary fig. S2*c*, Supplementary Material online). These quartets only implicate the internal branch around the four Asian pangolin species in corresponding colors in the main phylogeny. The value at the center of each quartet equals the proportion of gene trees following this quartet while the value to the right of the branch is the corresponding number of gene trees. Branches joining Carnivora to Pholidota have been shortened as indicated with two diagonal lines (//). Asterisks (*) indicate confiscated individuals whose origins could not be verified. Pangolin illustrations by Sheila McCabe.

Our partitioned concatenated (supermatrix), nonpartitioned concatenated, and coalescent (summary tree) phylogenies based on orthologous, whole-gene markers show robust support for the previously reported clear dichotomy between African and Asian pangolins. The three main clades are also in agreement with the three distinct pangolin genera (fig. 1 & supplementary fig. S2, Supplementary Material online; Gaubert et al. 2018). The seized individual from Sichuan China that was either identified as *M. culionensis* in Cao et al. (2021) based on mitogenomic data or identified as *M. crassicaudata* on NCBI (GCA_016801295) does not fit either classification. We find that it is a sister to the Southeast Asian pangolins (*M. javanica* and *M. culionensis*) rather than grouping with *M. culionensis* and does not group with the Indian pangolin (*M. crassicaudata*) either (fig. 1). Our full mitochondrial genome phylogeny supports the findings of the orthologous full nuclear gene phylogeny (supplementary fig. S3*a*, Supplementary Material online), while the Cytochrome b (*Cytb*; supplementary fig. S3*b*, Supplementary Material online) and Cytochrome c oxidase I (*COI*; supplementary fig. S3*c*, Supplementary Material online) gene phylogenies suggest that this individual forms a clade with two samples seized in Hong Kong. These two samples, with which this individual seized from Sichuan China groups, have been suggested to be a potentially novel Asian pangolin species based on *Cytb* and *COI* species delimitation (Zhang et al. 2015; Hu, Roos et al. 2020).

To determine whether this undescribed *Manis* species is a potential hybrid or at the least, an admixed individual with other Asian species, we performed f4-ratio, *f*-branch, and ancestry painting analyses across this group. The largest f4-ratio and *f*-branch ratio suggests that 4.91% of the genome common to the undescribed *Manis* sp. and *M. javanica* has been affected by gene flow (admixture proportion; supplementary fig. S4*a* and *b*, Supplementary Material online). The ancestral painting analysis confirmed low admixture of this species (supplementary fig. S4*c* and table S2, Supplementary Material online) as the proportion of heterozygous genotypes between two sets of putative parental combinations (*M. javanica* with *M. crassicaudata* or *M. culionensis*) were on average 1.9% (F1 generation hybrid would be ∼100%). Our findings suggest that the individual from Sichuan China is not a hybrid, but that there may have been low levels of gene flow between *M. javanica* and this *Manis* sp. in the past.

Our results using admixture, mitochondrial gene phylogenies, orthologous whole-gene phylogenies with high bootstrap support, and concordance analysis provide a suite of evidence that the individual seized from Sichuan China likely represents a separate Asian species (*Manis* sp.) rather than *M. crassicaudata* or *M. culionensis* (Cao et al. 2021). However, with high levels of cryptic diversity across pangolin species (Gaubert et al. 2016; 2018), notably within *M. culionensis* and *M. javanica* (Zhang et al. 2015; Nash et al. 2018; Hu, Roos et al. 2020), we cannot be certain that this previously undescribed taxon is indeed a new *Manis* species or part of a *M. javanica* species complex that includes this taxon and *M. culionensis*. Additional analyses using multiple geo-referenced wild samples for each species, as well as species boundary testing through genomic and morphological data will provide further insight into the status of this taxon and its distribution. These parameters will be crucial in determining conservation priorities and management plans for this potentially new species.

### Reticulation and Incomplete Lineage Sorting in Southeast Asian Pangolins

To assess the level of alternative topologies across the phylogenetic species tree, we used a gene (gCF) and site (sCF) concordance factor analysis, along with the proportion of alternative quartets around each branch of the main topology as obtained from the coalescent species tree analysis (Zhang et al. 2018; Minh, Hahn et al. 2020). Overall, there was a low level of discordance and proportion of alternative topologies (*d* = 0.981; fig. 1 and supplementary fig. S2*c*, Supplementary Material online), indicating a global robustness of the pangolin tree topology (Minh, Hahn et al. 2020). The clade containing *M. javanica* and *M. culionensis* had the highest values for both gene discordance (90.6 gCF/88.4 sCF) and alternative quartet topologies (8.81%; fig. 1 and supplementary fig. S2*c*, Supplementary Material online). *Manis javanica* and *M. culionensis* are currently regarded as distinct species based on five discriminant morphological characters (Gaubert and Antunes 2005) and phylogenetic species delimitation (Gaubert et al. 2018), although mean pairwise genetic distances between the two were lower than any other species combinations, including estimates across the six *P. tricuspis* lineages (Gaubert et al. 2018). Thus, our results demonstrate the need for extensive population-level estimates across the distributions of Southeast Asian pangolin species, including the undescribed *Manis* sp. taxon, to aid in species conservation planning and accurate postseizure repatriation of live individuals (Nash et al. 2018). The latter is particularly pertinent given the multiple Southeast Asian islands from which lineages of *M. javanica* are sourced and traded (Zhang et al. 2015; Nash et al. 2018).

**Fig. 2.**
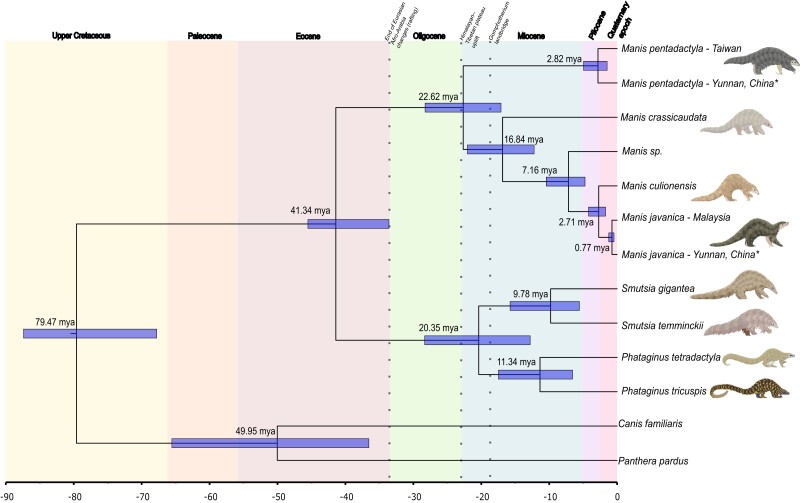
Time-calibrated phylogeny of extant pangolins. Mean posterior divergence times in millions of years and 95% Highest Posterior Density (HPD) intervals (nodal bars) for each node (see supplementary table S5, Supplementary Material online for values). The divergence estimates were based on the unpartitioned, IUPAC concatenated tree dataset of 58,724,014 bp (supplementary fig. S2*a*, Supplementary Material online) using Markov chain Monte Carlo (MCMC) sampling of 20 million generations (2 million generation burn-in) following an auto-correlated, log-normal relaxed clock model. We used fossil calibrations on the nodes of Ferae (upper bound from molecular data by Zhou, et al. (2011)), Carnivora and Pholidota as priors for the analysis (supplementary table S4, Supplementary Material online). Three geological/biological events of importance in our study are also highlighted by dotted lines and labels above the phylogeny. Asterisks (*) indicate confiscated individuals whose origins could not be verified. Pangolin illustrations by Sheila McCabe.

Given the relatively short branch length (as a result of recent divergence) possibly causing this uncertainty in separating *M. culionensis* from *M. javanica* through incomplete lineage sorting (ILS; Blom et al. 2016), we conducted a χ^2^ test on the gene concordance factors. The results suggest that ILS is the sole cause of discordance as the alternative topologies are not significantly independent (*P* < 0.05; supplementary table S3, Supplementary Material online) regarding the frequency of gene-trees supporting each topology (Huson et al. 2005; Zheng and Janke 2018). This is however not the case for the branch leading to the *M. crassicaudata*/*Manis* sp./*M. culionensis*/*M. javanica* clade whereby alternative topologies are significantly independent (fig. 1 and supplementary table S3, Supplementary Material online). The clade is also an outlier for the lowest site concordance (94.6 gCF/67.99 sCF), which points to site discrepancies for this clade. With introgression (ancient gene flow) and hybridization being possible causes of unbalanced gene-tree discordance of this clade (Doyle 1992), we tested for this across the clade using the aforementioned f4-ratio and *f-*branch statistics and across the entire topology using the maximum pseudolikelihood network reticulation analysis (Than et al. 2008). The results indicate limited admixture across the clade (supplementary fig. S4*a* and *b*, Supplementary Material online) and that a single reticulation event is the most likely scenario for pangolins (fig. 1). The timing and direction of this event is concurrent with that of the non-ILS related bias in alternative topologies determined by the χ^2^ analysis. The nonindependence caused by the higher alternative topology (bottom quartet; fig. 1) points to introgression/hybridization between a lineage sister-group to the African (contributing 31.9% of genes; supplementary fig. S5*a*, Supplementary Material online) or Asian (contributing 27.9% of genes; supplementary fig. S5*b*, Supplementary Material online) pangolin clades and the ancestor of Southeast Asian pangolins (including *Manis* sp.). The difference between the two potential basal lineage donors is due to whether we grouped all individuals from a species as the same species (basal Asian contributor), or kept them as separate evolutionary units (basal African contributor) for the reticulation analysis. Based on our average divergence estimates (fig. 2; see below), this event likely occurred during the Miocene (between 7.16 and 16.84 Ma).

### An Updated Biogeographic Scenario for Pangolins

Given the uncertainty of the basal branch contributor (Asian or African) in unison with the timing of the introgression event, we hypothesize that this ghost lineage was likely European. It may have moved toward Southeast Asia due to the shrinking of tropical environments in Europe during the middle Miocene climatic cooling (Jiménez-Moreno 2006), as seen with hominoids (Begun et al. 2012). This is plausible as pangolins are suggested to have occurred in Europe within the Miocene (*Necromanis*; Alba et al. 2018) and into the Pleistocene (*Smutsia olteniensis*, 2.2–1.9 Ma; Terhune et al. 2021). Alternatively, it may have been widespread in Laurasia and diversified throughout the region, before having gone extinct across parts of the continental mass. Additional population-based analyses and fossils across the region will aid in determining whether these hypotheses hold, and whether this ancient admixture event was adaptive (Figueiró et al. 2017). Recent fossils of pangolins found in South Africa (5 Ma, *S. gigantea*), India (Pleistocene, *M. lydekkeri*), and Java (42,000–47,000 ya, *M. paleojavanica*) provide further evidence of dramatic historical distribution changes (Gaudin et al. 2009; Terhune et al. 2021), and at the same time, that the potential effect of extinct lineages on our results needs to be considered.

To delve further into the scenario of pangolin diversification, the orthologous whole-genes were used to perform Bayesian estimation of divergence times guided by the coalescent tree and fossil calibrations (supplementary table S4, Supplementary Material online; dos Reis and Yang 2019). At most nodes, our genome-wide results concur with the divergence estimates and resultant biogeographic scenario of diversification previously described with fewer markers (fig. 2; supplementary table S5, Supplementary Material online; Gaubert et al. 2018; Hu, Roos et al. 2020). This is the case of the *Manis* sp. taxon which split from *M. culionensis* and *M. javanica* around the late Miocene (7.16 Ma; 4.73–10.42 Ma), similar to the times suggested by Hu, Roos et al. (2020) using *COI* and *Cytb* markers (6.95 Ma; 4.64–9.85 Ma).

We found that the two Southeast Asian pangolin species (*M. javanica* and *M. culionensis*) split during the Upper Miocene to Pliocene (mean = 2.71 Ma; 95% highest posterior density (HPD) = 1.70–4.21 Ma; supplementary table S5, Supplementary Material online), suggesting that the isolation by sea level rising (800–500 ka) of proto-Philippine pangolins coming from Borneo may have not been the original cause of their divergence (see: Gaubert and Antunes 2015). The split occurs around the same time as that between the *M. pentadactyla* population in Taiwan and the *M. pentadactyla* individual confiscated in Yunnan, China (2.82 Ma; 1.48–4.95 Ma), and overlaps with the split between the *M. javanica* population in Malaysia and the *M. javanica* individual confiscated in Yunnan, China (0.77 Ma; 0.46–1.24 Ma). These two novel, population-based estimates suggest divergences between lineages through relatively ancient population structuring for *M*. *javanica, M. culionensis,* and *M. pentadactyla* occurred before isolation of islands, as inferred in the deep, nearly species-level divergence of the two lineages of leopard cats (*Prionailurus bengalensis*) from Sundaland and mainland Southeast Asia (Luo et al. 2014). This is evidenced by the population divergence of *M. pentadactyla* having occurred even before the final separation of Taiwan from the continental mainland (Kawamura et al. 2016).

Our analysis indicates earlier divergence time estimates for Asian species than previously suggested (supplementary table S5, Supplementary Material online; Gaubert et al. 2018), particularly for the genus *Manis* (split between *M. pentadactyla* and the ancestor of *M. crassicaudata*/*Manis* sp*./M. culionensis*/*M. javanica*) which diverged during the Oligocene to Upper Miocene period (22.62 Ma; 17.07–28.23 Ma as opposed to 12.9 Ma; 10.3–15.6 Ma). The split of *M. pentadactyla* likely suggests a northern (*M. pentadactyla*; China)/southern (other *Manis* species; India, Indochina) Asian clade split, coincident with the Oligocene/Miocene boundary's second uplift of the Himalayan–Tibetan plateau in western China and extrusion of the Indochina block (Spicer et al. 2020; Deng et al. 2021). The biogeographic scenarios of other vertebrate species and current distribution limits of *M. javanica* and *M. pentadactyla* across the Ailao Shan-Red River shear zone (Tapponnier et al. 1990) coincide with this Indochina–China separation (Zhang et al. 2006; Che et al. 2010; Xiang et al. 2021). Overall, more fossil evidence is required to fully comprehend the evolution of pangolins given the paucity of the fossil record along with the possibility of multiple extinct lineages during the evolution of the group (Gebo and Rasmussen 1985; Gaudin et al. 2009). We also caution that our divergence estimates derive from complete nuclear genomes rather than mitochondrial genomes and nine nuclear markers, as well as the addition of a lower bound calibration for Pholidota (Gaubert et al. 2018), which could influence some of the observed differences between our estimates.

### Pangolin Demographic History is Shaped by Lineage-specific Biogeography

**Fig. 3.**
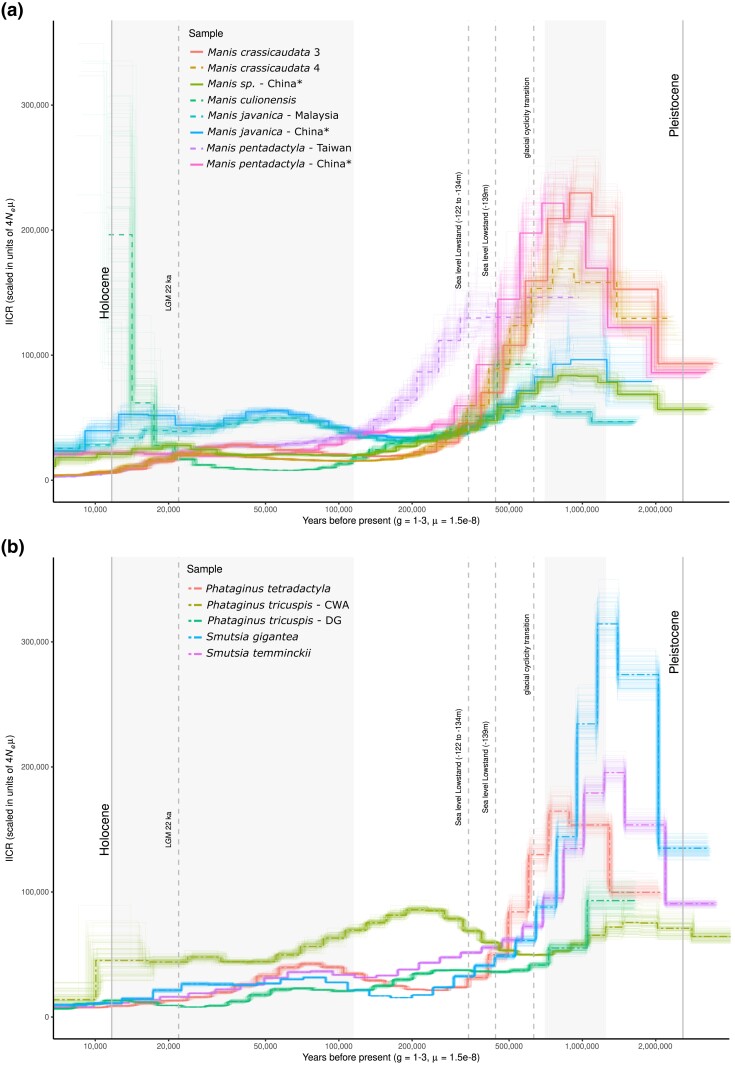
PSMC model on diploid genomes provide demographic trajectories of each pangolin species/population for the (*a*) Asian and (*b*) African continental clades. Bootstrap support of 100 iterations for each species/population are indicated by the lighter lines of the corresponding species/population principle line (thick single line). Approximate inverse instantaneous coalescence rate (IICR) values are indicative of effective population size (Ne), but may be influenced by changes in population size, connectivity and selection. Curves are scaled by a mutation rate of 1.5 × 10^−8^ substitutions per site per generation (µ) based on previous pangolin-related estimates (Choo, et al. 2016). Generation time in years (*g*) per species was estimated from available literature (supplementary table S7, Supplementary Material online). Vertical dotted lines with labels indicate important climatic events for this study while the shaded areas represent the Last Glacial Period (left) and Miocene-Pleistocene Transition (right). This figure was produced through a modified version of the following R script: https://github.com/elhumble/SHO_analysis_2020. Asterisks (*) indicate confiscated individuals whose origins could not be verified.

Although all species showed differing trends in inverse instantaneous coalescent rate (IICR) trajectories, most started to decline between 1,200 and 600 ka (pairwise sequentially Markovian coalescent [PSMC]; fig. 3) during the Mid-Pleistocene Transition (Chalk et al. 2017). This period relates to the transition from the less extreme shorter 41,000 year glacial-interglacial cyclicity to that of the more extreme 100,000 year cycles (Chalk et al. 2017), which may have led to population declines or structuring in pangolins as their habitats shifted in size and connectivity (Hewitt 2000; Palkopoulou et al. 2015). For the mainland Asian species/populations and *P. tetradactyla,* the most rapid phase of their declines occurred during a period that saw one of the two most extreme glaciation periods in the last 800 ka, the Marine Isotope Stage (MIS) 16 (676–621 ka; Lang and Wolff 2011).

Interestingly, the island species *M. culionensis* (Palawan Isl., Philippines) and the island population of *M. pentadactyla* (Taiwan) exhibited more recent declines (440–300 ka; fig. 3*a*) post-dating the last two major sea level lowstands of the past 500 ka (Rohling et al. 1998; Robles et al. 2015). These lowstand periods [440 ka (−139 meters compared to current sea levels) and 340 ka (−122 to −134 meters)] align with fossil and phylogeographic evidence of faunal migration to the current Asian islands during the Middle Pleistocene, including between the strait connecting China to Taiwan (c. 70 meters deep) and the strait connecting Borneo to Palawan (c. 145 meters deep; Tougard 2001; Hosoda et al. 2011; Kawamura et al. 2016; Ali 2018). It is, therefore, possible that after these lowstands, pangolins on these islands were isolated (population structuring) with a decreasing landmass (population decline) as sea levels rose. This also aided in the gene-flow of *M. javanica* (increased IICR) during the Last Glacial Period when sea levels fell (fig. 3*a*). This difference between island versus continental population fluctuations through time, and the influence of sea-levels for island populations has been found in Southeast island populations of *M. javanica* (Hu, Hao et al. 2020). The absence of such a signature in the two *M. crassicaudata* individuals from the island population of Sri Lanka could likely be due to the strait connecting India to Sri Lanka being only a minimum of 20 meters deep, allowing for extensive continental exchanges across the late quaternary climatic fluctuations (Bossuyt et al. 2004; Ali 2018). This links with the previously suggested nonmonophyly of Sri Lankan mitogenomes in the species (Gaubert et al. 2018).

**Fig. 4.**
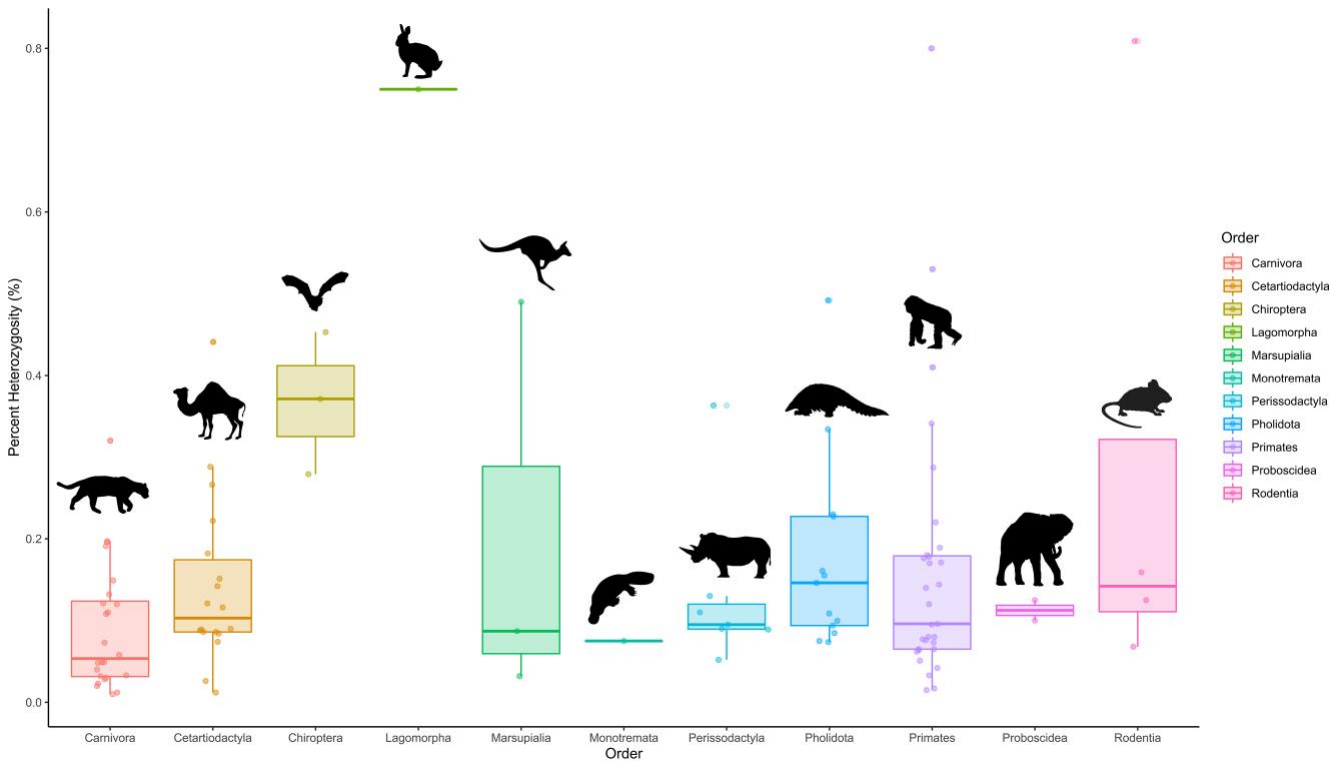
Proportion of genome-wide heterozygosity of pangolins (Pholidota) and other mammals. Estimates are grouped by mammalian order. Estimates are updated from the summary by Hu, Hao, et al. (2020), and displayed from smallest (least diverse) to largest (most diverse) by species in supplementary table S6, Supplementary Material online. Illustrations indicate the various taxonomic orders and are credited as follows (https://creativecommons.org/licenses/by/3.0/): Pholidota/Cetartiodactyla (Steven Traver), Carnivora (Gabriela Palomo-Munoz), Chiroptera (Margot Michaud), Lagomorpha/Marsupialia/Monotremata (Sarah Werning), Perissodactyla (Oscar Sanisidro), Primates (T. Michael Keesey), Proboscidea (Margot Michaud), Rodentia (Jiro Wada) sourced from PhyloPic (http://phylopic.org/).

The PSMC curves for the two *P. tricuspis* lineages that we tested showed differing trends; the Western Central Africa (WCA) lineage presented a stable IICR through time, while the Dahomey Gap (DG) lineage experienced a progressive decline (fig. 3*b*). These differences are mirrored by their genetic diversity whereby DG contains low levels of genetic diversity and high levels of inbreeding, while WCA has an opposite pattern (fig. 4, see below; Aguillon et al. 2020; Zanvo et al. 2022). Our differing results for the two *P. tricuspis* lineages (Gaubert et al. 2016) included in this study suggest that the demographic history of the species is likely complex, lineage specific, and will require more thorough investigation (notably since the DG sample had limited sequencing depth; supplementary table S1, Supplementary Material online). They also confirm that these two lineages have distinct evolutionary trajectories, which have been separated by strong biogeographic barriers (Gaubert et al. 2016), and thus should be treated as separated conservation units. Further deciphering of phylogeographic structure within pangolin species through population stratification methods is required before more complex demographic scenarios are assessed (Excoffier et al. 2021). Particularly since the PSMC's assumption of panmixia and its sensitivity to population structure strongly limit its interpretation (Salmona et al. 2017; Arredondo et al. 2021), especially in pangolins, which show high levels of cryptic diversity (Zhang et al. 2015; Gaubert et al. 2016; Nash et al. 2018).

### Delayed Loss in Genome-Wide Diversity

We estimated genome-wide heterozygosity using the site frequency spectrum inferred from genotype-likelihoods of full genome data (Korneliussen et al. 2014). We found large variation across pangolin species, up to a 6.65-fold divergence between the lowest and highest estimates (0.074–0.492% heterozygosity; fig. 4 and supplementary fig. S6, Supplementary Material online & supplementary table S6, Supplementary Material online). The *Manis* sp. individual (0.161%) has an above-average heterozygosity estimate for mammalian species (average = 0.156%; supplementary fig. S6, Supplementary Material online). This taxon is still, however, comparable to species of conservation importance like the silvery gibbon (*Hylobates moloch*; supplementary table S6, Supplementary Material online). The lower end of the heterozygosity spectrum is occupied by the *P. tricuspis* DG lineage (0.074%), one *M. crassicaudata* individual from Sri Lanka (MCR4; 0.075%), and *M. pentadactyla* (0.085%) from Taiwan. The latter population is from a deeply isolated island, suggesting that the founding effect, isolation from continental populations, and/or subsequent drift have reduced its levels of heterozygosity. This is a concern because long-term low population size and isolation can result in genetic load due to the accumulation and fixation of deleterious variants (Robinson et al. 2016). However, the major islands hosting pangolin populations (e.g., Sri Lanka, Taiwan, and Borneo) are large when compared to those iconic examples of such island effect (Robinson et al. 2016). Therefore, we believe that international trade, habitat fragmentation, and finite population structuring through geographical and anthropogenic barriers will probably play a larger role in decreasing heterozygosity levels of these island populations in the future, than their geographic isolation (Lane-deGraaf et al. 2014).

We hypothesize that the low heterozygosity of the *P. tricuspis* DG lineage can be related to the DG's bioclimatic history, which shaped a suboptimal savannah habitat intermixed with forest patches. Forest fragmentation through Holocene climatic oscillations (Salzmann and Hoelzmann 2005; Demenou et al. 2016) in conjunction with high levels of inbreeding through a steady IICR decline over the last 1 million years (fig. 3) may have resulted in a drastic decrease of effective population size and loss of heterozygosity, as noted using microsatellite data (Zanvo et al. 2022). Along with *M. culionensis* (0.492%), the other lineage of *P. tricuspis* (WCA; 0.334%) is an outlier for high levels of heterozygosity (fig. 4), alongside common species like the wild boar (*Sus scrofa*) and brown bear (*Ursus arctos*; supplementary table S6, Supplementary Material online). We nevertheless caution that our results may be influenced by genome quality or coverage (as is the case for *M. culionensis*; Mondol et al. 2013) and that they may not reflect species-wide diversity estimates as they are from only one or two representatives of each species. It has been shown that heterozygosity estimates can be heightened by admixture (Ackermann et al. 2019), however, we could not find interspecific admixture (supplementary fig. S4*a* and *b*, Supplementary Material online) that could have influenced the higher estimates found for *M. culionensis* (supplementary fig. S6, Supplementary Material online), providing further evidence of genome quality as a contributing factor. Additionally, nondegraded samples for *M. culionensis* are likely to provide lower heterozygosity estimates considering it is the most isolated species, found only on Palawan Island (Philippines) and surrounding satellite islands.

Overall, we suggest that conservation management plans should consider the genetic variability and demographic history within pangolin species, with special attention being placed on populations in suboptimal/fragmented habitats (DG) and islands (Taiwan, Palawan, and Sri Lanka). These heterozygosity estimates are likely not yet impacted by the recent boom in illicit pangolin trade (2016 for African species and a few pangolin generations earlier for Asian species), one of the biggest contributors to population declines (Mondol et al. 2013; Challender et al. 2020). We therefore suggest that the negative effects of recent population declines on heterozygosity may be noticeable in generations to come (Sovic et al. 2019). It will also worsen along with its deleterious consequences on the survival ability of pangolins if the global trafficking of pangolins remains uninterrupted. Such prediction further emphasizes the crucial role that the genomic monitoring of pangolin populations will have to play in the conservation of Pholidota in the near future.

### Providing Genomic Resources for Pangolin Conservation

In light of the drastic loss in genomic diversity likely to come (Dufresnes et al. 2018; van der Valk et al. 2019) and no evidence of a reduction in trade (Challender et al. 2020), the need for genomic data and genetic markers for pangolin conservation is evident. We, therefore, provide a database of 3,238 genes ranked by levels of mean pairwise identity (supplementary Database S1, Supplementary Material online: https://doi.org/10.5281/zenodo.7517409), which could be used to design forensic markers for trade monitoring and prosecution by providing evidence of possible geographic origin of poaching from seized samples (Zhang et al. 2015; Gaubert et al. 2018; Kotze et al. 2020; Ewart et al. 2021). Most genes have the ability to discriminate between, and likely within, the eight extant pangolin species, thus providing an opportunity to create typing tools using the most discriminative genes for forensic and population genomic inferences (e.g., SNP panels and mini-barcoding primers). After removing genes that we determined to be outliers (610 genes based on the mean and deviation of mean pairwise identity; supplementary fig. S7, Supplementary Material online), the database has 2,623 orthologous genes, totaling 3,410,610 segregating/polymorphic sites. The top gene (*TMEM38B*) has a mean pairwise identity of 79.51 ± 13.56% (min = 65.95%; max = 93.07%) and 20.86% parsimony informative sites. The outlier test is a conservative and basic measure of determining the top genes and so we provide the full list of genes for more in-depth forensic marker discovery. We also provide a good-quality reference genome for *S. gigantea* and the draft genomes of pangolin species for which virtually no current genomic data exist (many of which are geographically referenced). These genomic resources and the resultant inferences we made will be key for better conservation management, further conservation genetic research, and combating the illegal trade of pangolins (Allendorf et al. 2010; Kotze et al. 2020; Kardos 2021).

## Materials and Methods

### Sample Acquisition

Pangolin samples that were used for DNA extraction were collected in the field or from museum repositories (see supplementary table S1, Supplementary Material online for more information). Tissue samples were taken from two deceased *Manis crassicaudata* individuals who were confiscated from the trade in Sri Lanka. Tissue samples from *Phataginus tetradactyla* and *Smutsia gigantea* were collected from the Yaoundé bushmeat market, Cameroon. A spleen sample of *Smutsia temminckii* was collected from an individual who had succumbed to its injuries on an electrified game fence in the Kalahari Oryx Game Farm, South Africa. Finally, we collected a skin sample from *Manis culionensis* at the Field Museum of Natural History, Chicago (FMNH 62919), that had originated from Casuyan, Palawan Island, Philippines. Since the museum sample showed signs of degradation, we took precautions during the mapping and consequent filtering of its short-read data as well as interpretation of the results surrounding this species.

### Genome Sequencing and Assembly

#### Hybrid Assembly Reference Genome of the Giant Pangolin (*S. gigantea*)

High molecular weight DNA suitable for Oxford Nanopore Technologies (ONT) long-read sequencing was extracted from an ethanol-preserved muscle sample (CAM011) using the protocol optimized by Tilak et al. (2020). Long-read sequencing was performed with the ONT MinION instrument using libraries prepared with the ONT Ligation Sequencing kit SQK-LSK109 on four flow cells (FLO-MIN-106). This generated a total of 31.7 Gb of raw long-read data representing a genome depth-of-coverage of about 13x. ONT raw signal FAST5 files were then base-called with the high accuracy mode of Guppy v3.2.4 (https://github.com/nanoporetech) on a graphics processing unit (GPU) computing server of the Montpellier Bioinformatics Biodiversity platform (https://mbb.univ-montp2.fr/MBB) and subsequently cleaned using Porechop v0.2.3 (https://github.com/rrwick/Porechop). Complementary Illumina 150PE short reads were produced on an Illumina HiSeq 3000 sequencing system at the GeT-PlaGe sequencing platform at Genotoul (https://www.genotoul.fr). After cleaning using Trimmomatic v0.33 (options -phred33 LEADING:3 TRAILING:3 SLIDINGWINDOW:4:15 MINLEN:50; [Bolger et al. 2014]), we obtained about 185 Gb of short read data representing a genome depth-of-coverage of about 87 × (supplementary table S1, Supplementary Material online). A *de novo* hybrid genome assembly was performed using MaSuRCA v3.2.9 (Zimin et al. 2013), which first assembles short reads into “super-reads” before assembling them guided by the long ONT reads. To evaluate genome quality, traditional measures like the Benchmarking Universal Single-Copy Orthologs (BUSCO) score (Waterhouse et al. 2018), the number of scaffolds and contig N50, and mean and maximum lengths were computed.

*De novo* genome annotation was performed for our *S. gigantea* hybrid assembly and the DNA Zoo chromosome-length assembly of *P. tricuspis* following the approach used in Allio et al. (2021). First, repetitive elements were annotated and masked to avoid producing false evidence for gene annotations (Yandell and Ence 2012). This annotation was first performed for each genome independently using RepeatModeler v2.0.2 (Smit and Hubley 2008). To improve the accuracy of these *de novo* annotations, the libraries obtained from the different genomes were cleaned by removing protein-like sequences and were clustered for further analyses. The second step was to identify repeat elements by similarity search against publicly available libraries of mammalian repeats (Dfam [Wheeler et al. 2012]) using RepeatMasker v4.1.2-p1 (Tarailo-Graovac and Chen 2009). The annotations resulting from these two steps were synthesized in a General Feature Format (GFF) file to be fed into MAKER v3 (Holt and Yandell 2011).

To improve the gene annotation, we relied on transcriptomic information using publicly available RNAseq data for *M. javanica* (supplementary table S1, Supplementary Material online). To do so, raw reads from 25 transcriptomes were downloaded from Sequence Read Archive (SRA), cleaned using fastp v0.20.0 (Chen et al. 2018) using default parameters, and assembled with Trinity v2.9.0 (Grabherr et al. 2011). The resulting transcriptome assemblies were annotated with an adapted version of assembly2ORF (https://github.com/ellefeg/assembly2orf), which was specifically designed to annotate transcriptomes. This pipeline relies on evidence-based gene predictions to extract and annotate gene Coding DNA Sequences (CDSs) from transcriptome assemblies. The CDSs resulting from the annotation were concatenated and clustered by similarity with CD-HIT v4.8.1 (Li and Godzik 2006) to improve the efficiency of the subsequent MAKER annotation. The CDSs obtained from RNA-seq data were fed into MAKER v3 to help the evidence-based gene prediction. Additionally, the manually annotated, nonredundant protein sequence database Uniprot/Swiss-prot (Bairoch and Apweiler 2000; The UniProt Consortium 2009) was provided to MAKER v3 for the annotation.

To improve the annotation, three runs of MAKER v3 were performed iteratively. In the first run, evidence-based gene predictions using sequence similarities with the CDSs extracted from both the Swiss-prot database and the transcriptomes were computed. Then, two additional runs were performed, with SNAP (Single Nucleotide Polymorphism Annotation Platform) v2006-07-28 (Korf 2004) and Augustus v3.2.3 ((Stanke et al. 2006); via BUSCO v3, (Waterhouse et al. 2018)) as implemented within MAKER v3 to help create more sound gene models. In doing this, MAKER v3 uses the annotations from the two prediction programs in addition to the evidence-based gene predictions (similarities with reference CDSs) when constructing its models.

#### Draft Genomes for Other Pangolin Species

Paired-end Illumina short read (150 bp) sequencing was conducted on *Manis crassicaudata, M. culionensis, S. temminckii* and *P. tetradactyla* samples ranging from 44 × to 76 × sequencing depth (supplementary table S1, Supplementary Material online). SOAPdenovo2 vr240 (Luo et al. 2012) was used to assemble the genomes of all sequences generated in this study. The best kmer length assembly for each species was identified using SeqKit v0.9.3 (Shen et al. 2016) statistics on the scaffolds (N50, mean and maximum length) and the assemblies were gap-closed with GapCloser v1.12 (SOAPdenovo2).

#### Additional Genomic Data

Short read data from two previously published genomes of *M. javanica* and *M. pentadactyla* (Choo et al. 2016; Hu, Hao et al. 2020), an uncertain *Manis* species (labeled as *M. crassicaudata* on NCBI but published as *M. culionensis*) (Cao et al. 2021), and one current draft genome of *P. tricuspis* were extracted from the SRA on NCBI using the SRA toolkit v2.9.6 (supplementary table S1, Supplementary Material online; Leinonen et al. 2010). We also obtained chromosome-scale genome assemblies of *P. tricuspis* (https://www.dnazoo.org/assemblies/Phataginus_tricuspis; Choo et al. 2016) and *M. javanica* (https://www.dnazoo.org/assemblies/Manis_javanica) from DNA Zoo (Dudchenko et al. 2017, 2018). The former also included short read data that we used in our analyses (supplementary table S1, Supplementary Material online).

### Single-Copy Orthologous Gene Dataset

To build a comprehensive phylogenomic dataset for all extant pangolin species, we relied on the OrthoMaM v10 database (Scornavacca et al. 2019), which is composed of 14,509 single-copy orthologous genes for 116 mammal species, including 13,403 CDSs (coding DNA sequences) for *M. javanica*. We implemented a pipeline in which we used a dual strategy to reduce the effect on the high level of divergence between pangolin species (particularly between Asian and African clades) if only one reference was used. First, we used the *de novo* annotations to conduct single-copy orthologous gene extraction of the DNA Zoo reference genome assemblies for each pangolin clade (*Manis javanica* for Asian clade and *Phataginus tricuspis* for African clade). This provided us with what we termed reference CDSs and their gene identity codes (IDs). This was followed by subsequent mapping of Illumina reads of all species on their clade-specific reference full-genome assemblies and using the IDs from the CDS references to extract the relevant orthologous genes (supplementary fig. S1, Supplementary Material online).

#### CDS Extraction From *De Novo* Reference Assemblies

To extract the CDSs specifically corresponding to the single-copy orthologs of the OrthoMaM database, for each orthologous gene alignment, an hidden Markov model (HMM) profile was created via *hmmbuild* of the HMMER toolkit v3.1b2 (Eddy 2011). Then, all HMM profiles were concatenated and summarized using *hmmpress* to construct an HMM database. Finally, for each CDS newly annotated by MAKER v3, *hmmscan* was used on the HMM database to retrieve the best hits among the orthologous gene alignments. For each orthologous gene alignment, the most similar sequences for each species were detected via *hmmsearch*. Outputs from *hmmsearch* and *hmmscan* were discarded if the first hit score was not substantially better than the second (hit 2 < 0.9 hit 1). This ensured that our orthology predictions for the newly annotated CDSs were robust.

From this and previous work, we obtained orthologous CDS information from *Manis* (*M. javanica* from GenBank annotation and CDS extraction from OrthoMaM), *Phataginus* (*P. tricuspis* from DNAZoo, annotation and CDS extraction from this study), and *Smutsia* (*S. gigantea*, annotation and CDS extraction from this study).

#### Genome-Wide Mapping and Consensus

Short read data from all species were cleaned with fastp v0.19.4 with a base pair quality threshold (>20 Phred) and the paired-end base correction function using overlapped reads (-c option). The cleaned reads were then mapped to their respective clade-specific reference genome assemblies (Asian species: *M. javanica* from GenBank; *Phataginus*: *P. tricuspis* from DNA Zoo) using the default settings of the BWA-MEM (Burrows-Wheeler Aligner—maximum exact matches) algorithm of BWA v0.7.15 (Li and Durbin 2009) after testing mismatch, clipping and gap penalties. Due to the lower quality of the sequencing data of *Manis culionensis*, we applied a more stringent mapping approach for this species (seed = 23, mismatch penalty = 7). We used SAMtools v1.10 (Li et al. 2009) *view* to keep only reads mapped into a proper pair (-f 2) and mapped reads with a high mapping quality (>30 Phred). These filters were tested using SAMtools v1.10 flagstat before and after filtering, and using Qualimap 2 v0.7.1 (Okonechnikov et al. 2015) after filtering. Resulting bam files were sorted with SAMtools v1.10 *sort*, had duplicates marked with Picard v2.20.7 *MarkDuplicates*, as well as had genome-wide mean depth and proportion covered at a depth of >1 × and 10 × calculated using SAMtools v1.10 *depth* and a custom script (Custom script 1). ANGSD v0.933 (Korneliussen et al. 2014) *dofasta* was used to obtain consensus fasta sequences files for each bam file using genotype likelihoods (GL). Both option 3 (most common allele chosen to make haploid/haplotype consensus) and option 4 (multiple alleles chosen to make IUPAC consensus) were chosen in conjunction with command *docounts* 1. Reads were filtered if they were of bad quality (-remove_bads), not paired (-only_proper_pairs), below a mapping quality of Phred 30 (-minmapq), and had multiple mappings (-uniqueOnly). We also filtered for bases below Phred 20 (-minQ; except *M. culionensis* which had Phred 30) and a minimum sequence depth of 10 × (-setMinDepth) and a maximum of two times that of the average depth per individual (-setMaxDepth). Outgroup taxa *Panthera pardus* (GCF_001857705.1) (Kim et al. 2016) and *Canis familiaris* (https://www.dnazoo.org/assemblies/Canis_lupus_familiaris_Basenji), which are part of the sister order Carnivora (Liu et al. 2017), underwent the same process with each being mapped to their respective Hi-C reference genomes from DNAZoo (Dudchenko et al. 2017, 2018).

#### Obtaining Orthologous Full Gene Markers Using Orthologous CDS IDs

Using the orthologous CDS gene IDs from OrthoMaM for each pangolin reference (*M. javanica* and *P. tricuspis*), whole-gene annotations containing these IDs were extracted from the annotation files of the references (*M. javanica*, *P. tricuspis* and the two outgroup species; Custom script 2). Annotations with duplicates and those that were not found in both pangolin references were removed. This left us with gff3 annotation files of 5,660 orthologous full genes. BEDtools v2.29.0 (Quinlan and Hall 2010) *getfasta* was used to extract these orthologous full genes from the full genome IUPAC and haploid/haplotype consensuses, after being indexed with SAMtools v1.10 *faidx*.

### Phylogenomics and Diagnostic Markers From Genes

Multigene fasta files of each species were converted into multispecies fasta files per gene marker using SeqKit v0.9.3 (Shen et al. 2016) *split* (−by-id option) and subsequent concatenation using the *cat* command. These consisted of all eight species (including two representatives each of *M. javanica* and *M. pentadactyla*) and the outgroup taxa from the Order Carnivora. The dataset was then split into two parts, one with haplotype (haploid) consensus genes of only one representative of the eight known species, and the second with IUPAC consensus genes of all aforementioned pangolin individuals and the outgroup taxa. The first was used to determine per-gene diversity estimates across pangolins and the second was used for phylogenetic testing, divergence time estimation, and identifying reticulation events.

#### Genetic Diversity Estimates From Whole Genes

Obtaining genetic diversity estimates for pangolins from genes requires the use of one representative per species and the removal of outgroup taxa as these would bias the level of diversity per gene. Hence, the two Carnivora outgroup taxa, together with *M. javanica, M. pentadactyla,* and *Manis* sp. [from Choo et al. 2016; Cao et al. 2021] were removed from the gene dataset before undergoing alignment. Sequences were aligned using MAFFT (Multiple Alignment using Fast Fourier Transform) v7.313 (Katoh and Standley 2013) –auto option and transitive consistency score (TCS) (Chang et al. 2014) for each alignment were assessed using T-COFFEE v11.00.8 (Notredame et al. 2000) *evaluate* with the clustalw2_msa method. Multiple sequence alignments (MSAs) with TCS scores under 80 were removed based on the possibility of paralogy, repetitive regions, or mis-annotated genes between the two pangolin reference genomes. This resulted in 3,238 gene MSAs of high quality for further analyses. PhyKIT v1.1.3 (Steenwyk et al. 2021) was used to obtain statistics on the pairwise proportional identity of each marker (command *pairwise_identity*) and parsimony informative sites (command *parsimony_informative_sites*). Due to PhyKit calculating alignment gaps as informative sites, all gaps were removed before this analysis using TrimAL v1.4.1 (Capella-Gutiérrez et al. 2009) with the option –nogaps. DnaSP v6 (Rozas et al. 2017) was used to conduct the “DNA Polymorphism” analysis in batch mode to obtain the rest of the diversity estimates. These outputs were then merged with a custom script (Custom script 3) to obtain a range of diversity statistics per gene. Using the package “robustbase” v0.93 (Maechler et al. 2021) in R v3.6.1 (Rstudio Inc., Massachusetts, USA), we identified possible outlying genes with low levels of mean proportional pairwise identity (or high levels of diversity between species) by filtering values over two Qn deviations from the median of both the mean (487 outliers) and standard deviations (566 outliers) of pairwise identities (supplementary fig. S7, Supplementary Material online) (Rousseeuw and Croux 1993). This was done to prevent inconsistent markers from being used in forensic screening approaches due in part to possible biological reasons (paralogy, repetitive elements, inconsistency across taxa, etc.).

#### Phylogenomic Tree Building and Concordance Analyses

From the results on TCS alignments scores above, the same 3,238 cleaned, orthologous genes with all pangolin individuals and outgroup taxa were aligned and trimmed following the aforementioned protocol (MAFFT v7.313 and TrimAL v1.4.1), however, the –gappyout option was used for trimming. Additionally, these alignments stem from IUPAC consensus and they were again evaluated with T-COFFEE v11.00.8 in order to remove outgroup taxa that had a TCS score of lower than 90 (using SeqKit v0.9.3 *grep*). Alignment statistics of each MSA file per gene were obtained and were then concatenated into a single MSA using AMAS (Alignment Manipulation And Summary) v0.98 *summary* and *concat* (Borowiec 2016) with –part-format set to RAxML in order to provide a partitioning file by gene. ModelTest-NG v0.1.5 (Darriba et al. 2019) was used to identify the best model of sequence evolution for each gene-partition (input partition file from AMAS) as well as the entire alignment (no partition). We set the -h option to *uigf* (tests rate heterogeneity: Uniform, +I, +G, and +I & +G) and -T option (model test template) to *phyml* (all 11 models of evolution) in order to test 88 DNA models. Partitioned and nonpartitioned concatenated IUPAC phylogenies (supermatrix) with 1,000 Felsenstein bootstrap replicates were inferred from the concatenated MSAs using RAxML-NG v0.9.0 (Kozlov et al. 2019) following the best-fitted DNA model for each partition under the Bayesian information criterion (BIC) test. A multiple species coalescent summary tree was also inferred from the MSAs by first using RAxML-NG v0.9.0 to build a gene tree per gene whilst accounting for the best-fitting DNA model (BIC) for each gene (extracted from the aforementioned ModelTest-NG analysis), followed by summarizing these gene trees with ASTRAL-III v5.6.3 (Zhang et al. 2018). A polytomy test (-t10) using these data was conducted in order to test whether each branch is a potential polytomy (Sayyari and Mirarab 2018), but none were suggested as a polytomy. Finally, gene (−gcf) and site (−scf with 100 quartets randomly sampled around each internal branch) concordance factor analyses based on the aforementioned gene trees and ASTRAL-III output (coalescent species tree) were performed with IQ-TREE 2 v2.0.6 (Minh, Schmidt et al. 2020). This provides a full analysis of the raw data in terms of genes or sites that may be disagreeing with the species tree phylogeny. This was followed by a χ^2^ test of independence in R v3.6.1 (Rstudio Inc., Massachusetts, USA) based on a script designed by Robert Lanfear (http://www.robertlanfear.com/blog/files/concordance_factors.html). This tests the frequencies of two alternative quartet topologies of each internal branch whereby significance indicates the possibility of something other than ILS causing gene-tree discordance. We did not use this test on site concordance frequencies, as this test does not take linkage disequilibrium between sites into account, which can be assumed to play a large role for multiple sites in a single gene.

#### Reticulate Evolutionary Relationships

We used PhyloNet v3.8.2 (Than et al. 2008) to run two separate analyses in order to determine possible reticulation events through maximum pseudolikelihood estimates. The first was to consider each individual as a separate species and the second was to merge multiple individuals of the same species as one. Both analyses were conducted by inputting the RAxML gene trees whilst the best number of reticulation events (0–4) was determined by comparing the change in maximum pseudolikelihood scores between them. PhyloPlot in Julia (Solís-Lemus et al. 2017) was used to visualize the networks.

#### Divergence Time Estimates

We used MCMCTree in the PAML-4.9 h package (Yang 2007) to estimate divergence times on the nonpartitioned RAxML-NG (supermatrix) phylogeny inferred from the IUPAC consensus gene dataset. We used Ferae (root), Pholidota, and Carnivora fossils as upper and lower-bound calibrations (supplementary table S4, Supplementary Material online). We followed dos Reis and Yang (2019) by first identifying the substitution rate (rgene gamma) for the IUPAC gene dataset using baseml and then doing the approximate likelihood calculation by obtaining gradient (g) and Hessian (H) values for branch lengths. The Markov chain Monte Carlo (MCMC) sampling from the posterior distribution of times and rates using MCMCTree was run for 20 million generations with a burn-in of 2 million generations and was run twice to test for convergence in Tracer from BEAST 2 (Bouckaert et al. 2014). An auto-correlated, log-normal relaxed clock model was implemented. Finally, we ran the analysis by sampling from the prior of times and rates (usedata = 0) to test the soundness of the prior and whether our calibrations may influence the results.

### Mitochondrial Genes and Genomes

To obtain mitochondrial genomes of each species in this study as well as those from the undetermined *Manis* sp. (Cao et al. 2021) and the two unpublished *P. tricuspis* reference genomes, 10 million reads were extracted from fastq files and mapped to a published mitochondrial genome reference of each species using the default options of Geneious mapper in Geneious v9.1.8 (Kearse et al. 2012). These were then consensus called and aligned to the mitochondrial genome dataset from Gaubert et al. (2018) with MUSCLE (ten iterations) in Geneious. The alignment underwent Neighbor-Joining (NJ) phylogeny testing in MEGA X (Kumar et al. 2018) with Kimura two-parameter model of evolution and 1,000 bootstrap replicates. We also aligned the *COI* and *Cytb* genes from two samples of the potential new Asian species suggested by Hu, Roos et al. (2020) to the mitochondrial genome dataset and extracted these regions for an additional NJ phylogeny of these two genes.

### Genome-Wide Analyses

The aforementioned cleaned short read data for each pangolin species were mapped to their closest reference per genus (*Phataginus*: *P. tricuspis* DNA Zoo; *Manis*: *M. javanica* DNA Zoo; *Smutsia*: *S. gigantea* this study) using BWA-MEM from BWA v0.7.15. Resulting bam files were filtered using SAMtools v1.10 *view* (-f 2; > 30 Phred), sorted with SAMtools v1.10 *sort*, had duplicates marked with Picard v2.20.7 *MarkDuplicates*, as well as had genome-wide mean depth and proportion covered at a depth of >1 × and 10 × calculated using SAMtools v1.10 *depth* and a custom script (Custom script 1).

#### Comparative Genome-Wide Heterozygosity

ANGSD v0.933 (Korneliussen et al. 2014) was used to estimate genome-wide heterozygosity per sample using a script adapted from de Jager et al. (2021) following the ANGSD workflow (http://www.popgen.dk/angsd/index.php/Heterozygosity). To do this, the command *doSaf* was implemented on the alignment bam file of each individual from genome-wide mapping to infer its folded site allele frequency likelihood. We used the genus-specific reference as the ancestral state and the SAMtools method for -GL 1 as it considers possible sequencing errors and performs necessary corrections. The option -C 50 was used to adjust mapping quality for excessive mismatches, and reads were removed if they were of bad quality (-remove_bads), not paired (-only_proper_pairs), below a mapping quality of Phred 30 (-minmapq), and had multiple mappings (-uniqueOnly). Bases below Phred 20 were also removed (-minQ), with the exception of *M. culionensis* which was increased to Phred 30 due to genome quality. After *doSaf*, we inferred the site frequency spectrum with the ANGSD subprogram realSFS, and finally, the genome-wide heterozygosity by dividing the number of heterozygous sites by the total number of sites per genome. We extended the list of genome-wide heterozygosity estimates for mammalian species built by Hu, Hao et al. (2020), many of which are of conservation importance, by including our estimates as well as additional estimates published since the conception of the table.

#### Demographic History Reconstruction With PSMC

IICR trajectories (Arredondo et al. 2021), which can be related to fluctuations in effective population size and connectivity, were estimated from the whole-genome mapping data of each pangolin species using the PSMC model (Li and Durbin 2011).

Before this could be done, each alignment file (bam) from the whole-genome pipeline output was converted to a diploid consensus fastq file by first determining Genotype Likliehoods (GL) using BCFtools v1.8 *mpileup* (minimum base and mapping quality of Phred 30), calling the genotypes with BCFtools v1.8 *call* (-c option for consensus caller), and then filtering and converting the Variant Call Format (VCF) to a fastq file using the vcftultils.pl vcf2fq script (SAMtools v1.10). Filtering with vcfutils.pl included a minimum mapping quality of Phred 30 (-Q), a minimum coverage threshold of 10 × (-d) and maximum coverage threshold of double the mean genome-wide coverage of each individual (-D) as determined from the whole-genome pipeline. For all genomes, the mean genome-wide depth was higher than the recommended PSMC cutoff of ≥18 × and the proportion of missing data per genome at ≥10 × depth was lower than the recommended PSMC cutoff of 25% (Nadachowska-Brzyska et al. 2016). This was the case except for one of the two *M. crassicaudata* (MCras 4; depth = 9 × and 37% of reference covered at ≥10×) and the *P. tricuspis* (From DNA Zoo; depth = 10.8 × and 45% of reference covered at ≥10×) genomes. These were only used for PSMC and heterozygosity estimates and should be taken with caution.

Using the program PSMC v0.6.5 (Li and Durbin 2011), the fastq consensus files with diploid variant information were then converted to fasta-like files (psmcfa) containing information of whether there is at least one homozygote in a bin of 100 bp. From there, we ran the PSMC analysis following the default settings described by Li and Durbin (2011) for human populations (-N 25, -t 15, -r 5, -*P* “4 + 25*2 + 4 + 6”) and used on other mammalian species (Westbury et al. 2018; Chen et al. 2019; de Jager et al. 2021). By randomly sampling subsections of the psmcfa file, 100 bootstrap replicate analyses were performed in order to estimate the variance in the approximate inverse instantaneous coalescence rate (IICR). This is the inverse of the rate at which coalescence events take place through time, which was originally used as a function of effective population size in a panmitic model (Li and Durbin 2011). However, inferences must take into account the potential of a nonpanmitic model of demographic history and the confounding effects of natural selection and population structure, which can also affect coalescence (Arredondo et al. 2021; Johri et al. 2021). A mutation rate of 1.5 × 10^−8^ years/site was implemented (Choo et al. 2016), while a generation time in years was chosen for each species depending on available literature (supplementary table S7, Supplementary Material online). The PSMC figures combining the species from each continental clade were constructed in R v5.1 (Rstudio Inc., Massachusetts, USA) following de Jager et al. (2021) by editing a script (found at: https://github.com/elhumble/SHO_analysis_2020) that utilizes the plotPsmcR function (Liu and Hansen 2017).

#### Tests of Admixture

To test if admixture or the possibility of hybrids could have played a role in the identification of the undescribed *Manis* sp., we tested whether Asian species showed levels of admixture, particularly with the *Manis* sp. individual.

We used BCFtools v1.8 to obtain genotypes (*mpileup, call*, and *norm*) from the Asian species alignment files (bams), and filtered these genotypes by depth (10 × to two times average depth per species), and site and mapping (>40 Phred) quality (Danecek et al. 2011, 2021). The multisample filtered VCF was then used to obtain f4-ratio (proportion of genome affected by gene flow – admixture proportion) and *f-*branch (uncorrelated admixture proportion across the tree) statistics with Dsuite v0.4 r38 (Malinsky et al. 2021). Here, *M. pentadactyla* was placed as the outgroup for the trios tests and the phylogeny from figure 1 (partitioned concatenated IUPAC phylogeny from RAxML) was used as the tree input. Scripts (get_fixed_site_gts.rb and plot_fixed_site_gts.rb) within Dsuite were used to undertake ancestry painting (genotypes carried at sites that are fixed between the presumed parental species) whereby *Manis* sp. and *M. culionensis* were proposed as the putative hybrid to various putative Asian species combinations (see: supplementary table S2, Supplementary Material online for the trio combinations).

## Supplementary Material

msad190_Supplementary_Data

## Data Availability

Draft genomes (*Manis culionensis, M. crassicaudata, Phataginus tetradactyla, Smutsia temminckii*) and the hybrid assembled, annotated reference genome with associated metadata (*S. gigantea*) are available in the GenBank Nucleotide Database (BioProject: PRJNA795390). The associated sequence read data have also been deposited in Genbank (SRA: SRR17702824-SRR17702828) for the aforementioned genomes (except for *S. temminckii*). The accession numbers or links for all accessed genomic data are listed in supplementary table S1, Supplementary Material online. A database containing the list genes ranked by diversity amongst all eight pangolin species has been deposited at Zenodo and is publicly available (supplementary Database S1, Supplementary Material online: https://doi.org/10.5281/zenodo.7517409). All original code in the form of custom scripts for processing the genomics data in this study have also been deposited at Zenodo and are publicly available (Custom scripts 1–3: https://doi.org/10.5281/zenodo.7517409). Any additional information required to reanalyze the data reported in this paper is available from the lead contact upon request.
