# Supplementary material for: Pangolin Genomes Offer Key Insights and Resources for the World’s Most Trafficked Wild Mammals
Source: Mol Biol Evol. 2023 Oct 5;40(10):msad190. doi: 10.1093/molbev/msad190 (PMC10551234; doi:10.1093/molbev/msad190)
Supplement: msad190_Supplementary_Data [file msad190_supplementary_data.docx]

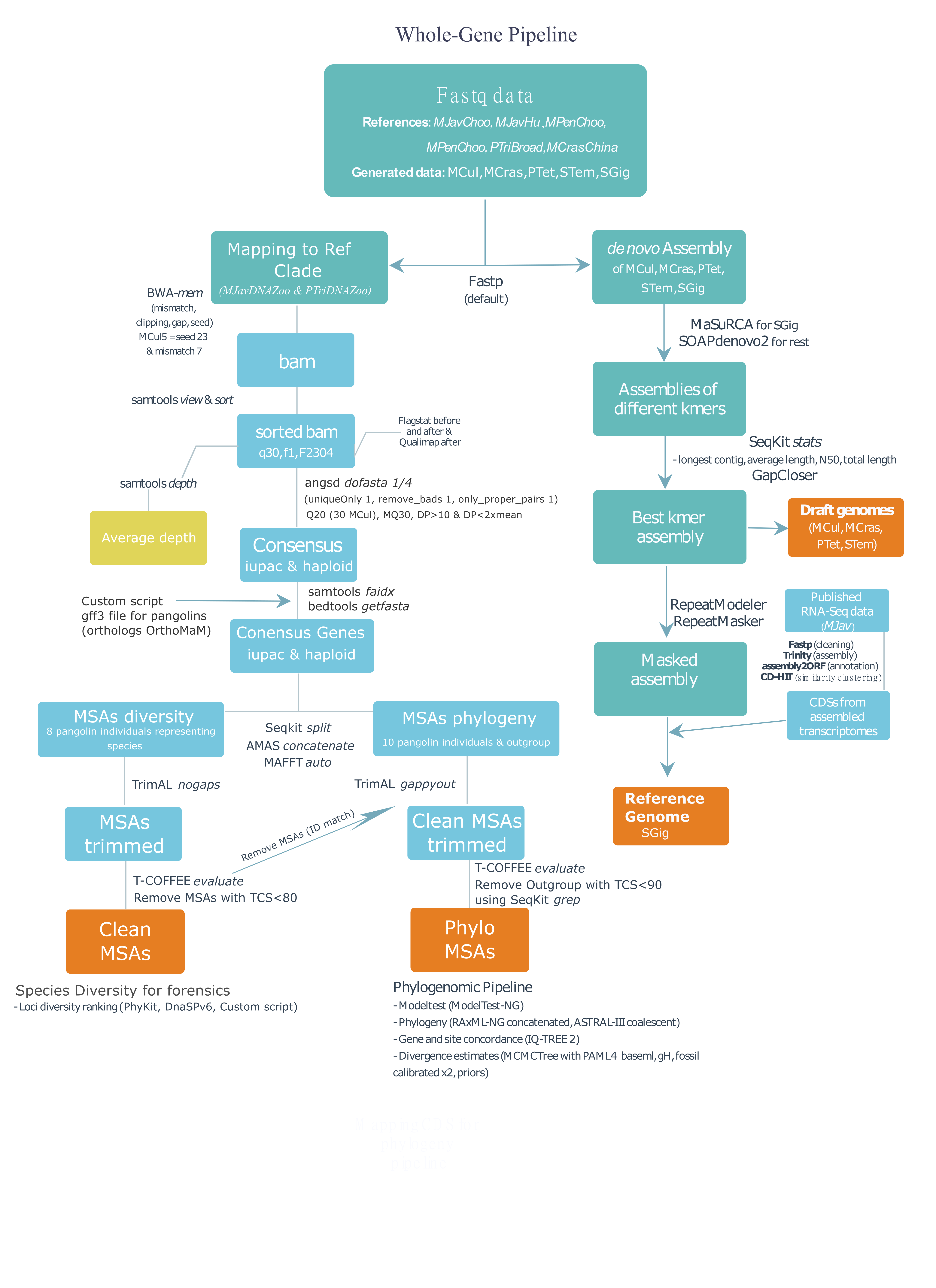


**Figure S1:** Orthologous whole-gene marker pipeline and subsequent analyses. This pipeline was formulated and used to create the whole-gene markers (introns and exons) required for further analyses. During this process, the reference genome (*Smutsia gigantea*) and draft genomes for four other pangolin species were created. First, the *de novo* assembly side of the pipeline (right side) was conducted before the whole genome and subsequent whole-gene extraction side of the pipeline could be conducted (left side). Custom scripts can be found at Zenodo (https://doi.org/10.5281/zenodo.7517409). We used shorthand notations to indicate the genomes used for each species as follows: MJavChoo - *Manis javanica* from Malaysia (Choo, et al. 2016), MJavHu – *M. javanica* confiscated in China (Hu, Hao, et al. 2020), MPenChoo – *Manis pentadactyla* from Taiwan (Choo, et al. 2016), MPenHu – *M. pentadactyla* confiscated in China (Hu, Hao, et al. 2020), MCrasChina – *Manis sp.* confiscated in China (Cao, et al. 2021), PTriBROAD – *Phataginus tricuspis* (unpublished BROAD institute; GCA_004765945.1), PTriDNAZoo – *P. tricuspis* (unpublished DNA Zoo; <https://www.dnazoo.org/assemblies/Phataginus_tricuspis>), MCul – *M. culionensis* (this study), MCras – *M. crassicaudata* (this study), PTet – *P. tetradactyla* (this study), SGig – *Smutsia gigantea* (this study), STem – *S. temminckii* (this study). Further information on these genomes and samples can be found in Table S1.


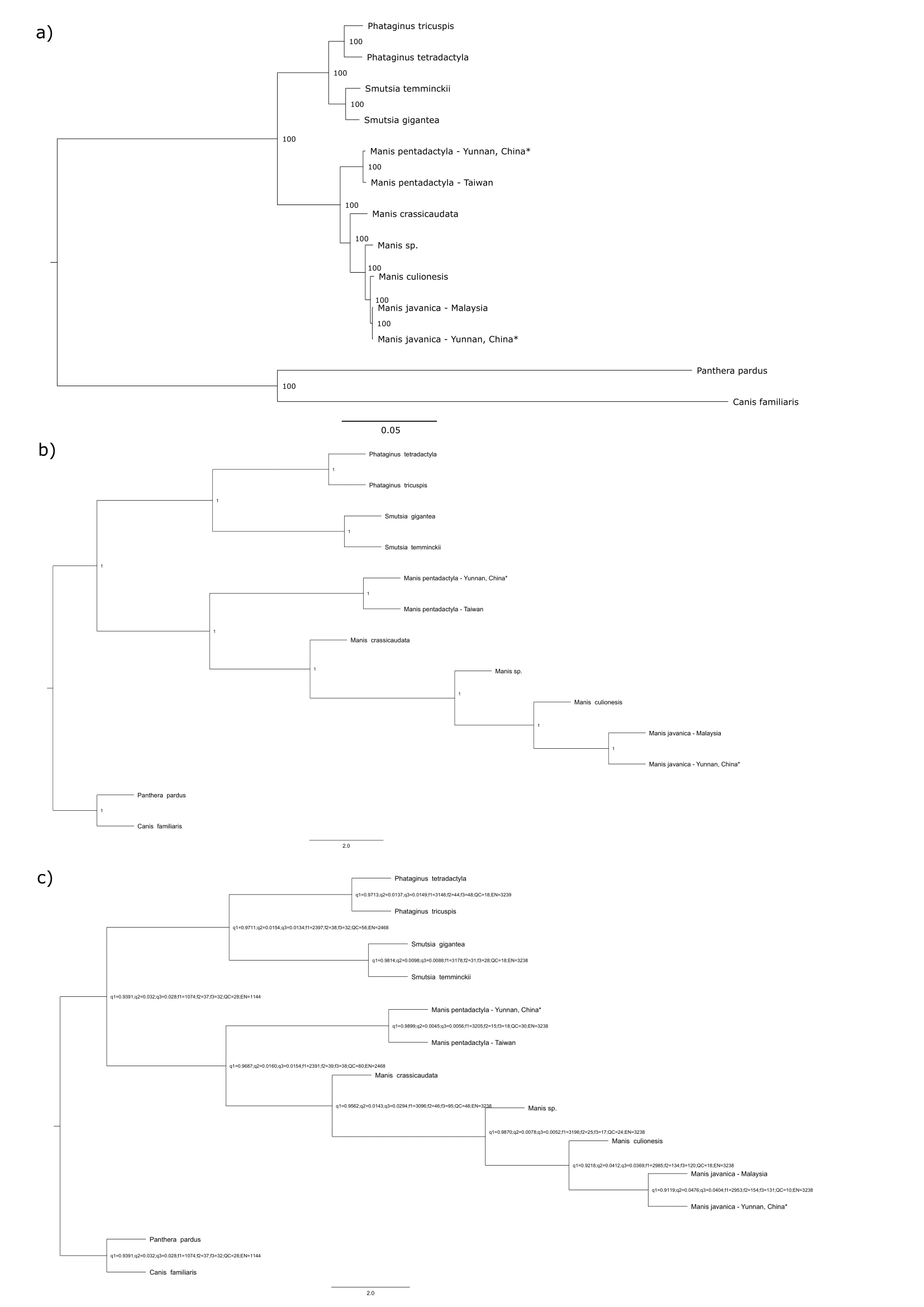


**Figure S2:** Phylogenomic relationships of pangolins inferred from concatenated and coalescent trees.

1. Non-partitioned concatenated phylogeny based on 58 724 014 bp from 2 238 IUPAC consensus whole-gene markers. The pangolin phylogeny consists of 13 individuals from all eight species and is rooted with two representatives of the sister Order Carnivora (*Canis familiaris* and *Panthera pardus*). Nodal values indicate bootstrap support from 1 000 Felsenstein replicates. The model used for the maximum likelihood tree search was the TVM+FO+G4m based on a best-fit DNA model test.
2. Multiple species coalescent phylogeny based on 2 238 gene trees. Branch lengths are in coalescent units while nodal values indicate local posterior probability support (1=complete support).
3. Multiple species coalescent phylogeny based on 2 238 gene trees. Branch lengths are in coalescent units. Nodal values indicate the level of gene tree conflict, which is calculated through the number of alternative gene tree quartets that agree with the main species tree quartet topology (this figure) at each internal branch. The normalized quartet score for the entire phylogeny is 0.981. Using a polytomy test we did not identify any polytomies in the tree (which could influence these results). q1, q2, and q3 refer to the proportion of quartets in the gene trees that agree with a branch (quartet support) for the main topology (LR|SO), first alternative (RS|LO) and second alternative (RO|LS), respectively. f1, f2, and f3 refer to the same as above but are the raw number of quartet trees instead of the proportion. QC is the total number of quartets possible around each branch and EN is the number of effective genes for each branch.

Asterisks (*) indicate confiscated individuals whose origins could not be verified.





**Figure S3:** Phylogenetic relationships of pangolins inferred from mitochondrial gene and genome trees.

1. Full mitochondrial genome phylogeny based on 16 437 bp using the Neighbor-Joining method in MEGA X (Kumar, et al. 2018). The pangolin phylogeny consists of 26 individuals from all eight species and six cryptic lineages of *Phataginus tricuspis*. Nodal values relate to bootstrap support (1 000 replicates). The DNA model of evolution was Kimura 2-parameter with all positions with <90% site coverage eliminated (partial deletion option).
2. Full cytochrome b (*Cytb*) gene phylogeny based on 399 bp using the Neighbor-Joining method in MEGA X (Kumar, et al. 2018). The pangolin phylogeny consists of 28 individuals, including that from all eight species, the six cryptic lineages of *Phataginus tricuspis,* and the two samples suggested as a possibly new *Manis* species (Hu, Roos, et al. 2020). Nodal values relate to bootstrap support (1000 replicates). The DNA model of evolution was Kimura 2-parameter with all positions with <90% site coverage eliminated (partial deletion option).
3. Full cytochrome oxidase subunit I (*COI*) gene phylogeny based on 600 bp using the Neighbor-Joining method in MEGA X (Kumar, et al. 2018). The pangolin phylogeny consists of 28 individuals, including that from all eight species, the six cryptic lineages of *Phataginus tricuspis,* and the two samples suggested as a possibly new *Manis* species (Hu, Roos, et al. 2020). Nodal values relate to bootstrap support (1000 replicates). The DNA model of evolution was Kimura 2-parameter with all positions with <90% site coverage eliminated (partial deletion option).


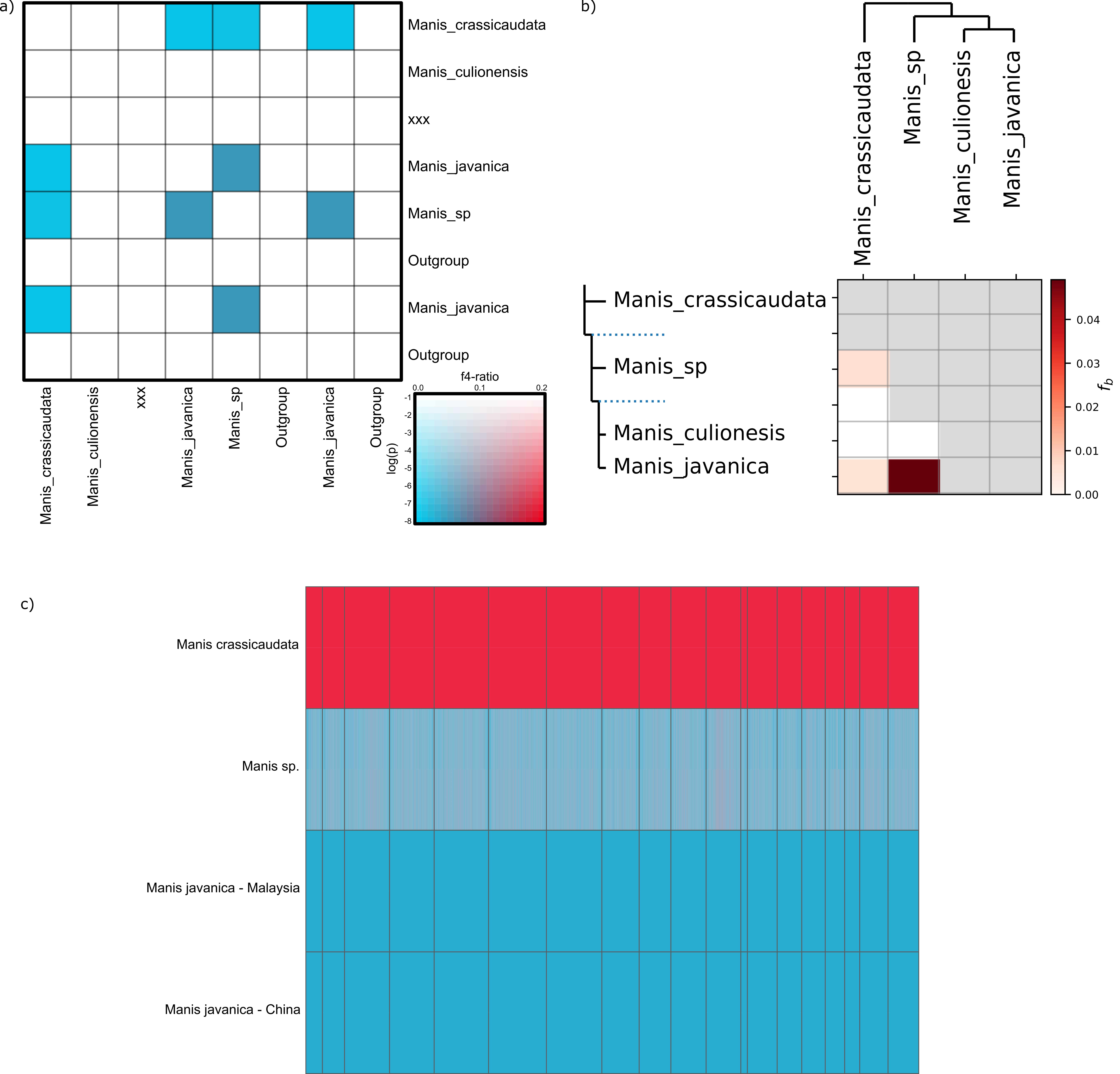


**Figure S4:** Tests of admixture and hybridization between Asian pangolin species. (a) Heatmap of f4-ratio statistics whereby warmer colours indicate higher admixture proportions. The ‘Outgroup’ refers to *Manis pentadactyla* individuals whilst ‘XXX’ refers to samples left out of the analysis. (b) Heatmap of *f-*branch statistics (uncorrelated f4-ratios) whereby warmer colours indicate higher the admixture proportions. Grey shaded areas represent non-testable combinations. The tree on each side represents the phylogenetic relationships between species whereby *Manis pentadactyla* is not included since it was set as the outgroup species. (c) Ancestry painting plot to test for hybrid species whereby the undescribed *Manis* sp. was set as the putative hybrid species of the two putative parental species: *Manis crassicaudata* (red) and *Manis javanica* (blue). The bottom row of the *Manis* sp. sample column is coloured in red and the top row is coloured in blue when the genotype is heterozygous. The proportion of heterozygous genotypes is 0.02 whilst an F1 hybrid would be close to 1.0 (see Table S2 for other parental-hybrid combinations).


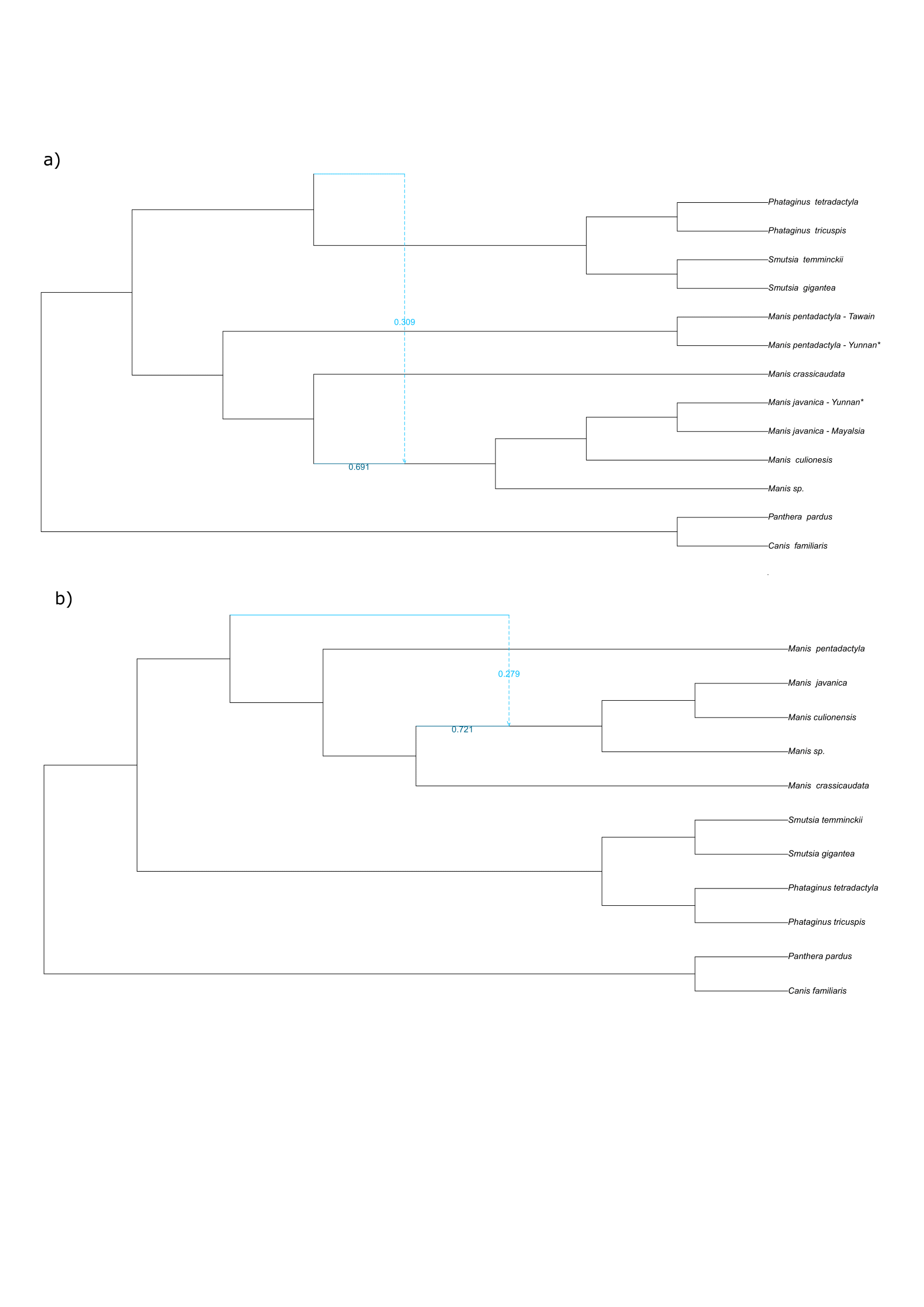


**Figure S5:** Phylogenetic networks using maximum pseudolikelihood estimates, depicted as rooted phylogenies to infer reticulation (introgression/hybridization) events within pangolins. Dotted blue lines indicate connection and direction of gene-flow between the donor and recipient taxa. Solid blue lines indicate ancestry of donor and recipient taxa. Numbers correspond to the proportion of genes shared between recipient and donor (light blue) and recipient and ancestor (dark blue). Analyses were run twice, (a) first by using each individual as a spate evolutionary unit and then (b) by indicating that multiple individuals of the same species were the same species. Both analyses indicated one reticulation event as the most likely outcome. Networks were drawn using the PhyloPlot function in Julia. Asterisks (*) indicate confiscated individuals whose origins could not be verified.


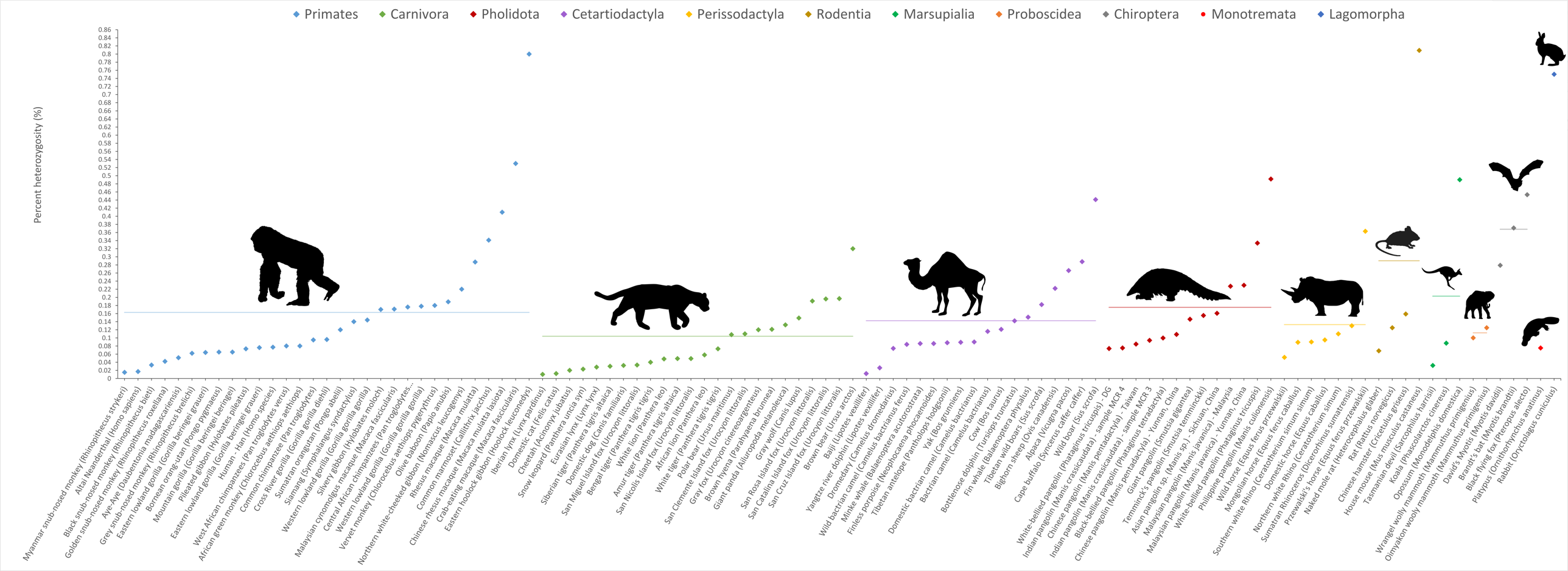


**Figure S6:** Proportion of genome-wide heterozygosity of pangolins (Pholidota) and other mammals. Estimates are grouped and color-coded by mammalian order with the horizontal line indicating the average proportion of heterozygosity for each order. Estimates are updated from the summary by Hu, Hao, et al. (2020), and displayed from smallest (least diverse) to largest (most diverse) in Table S6. Illustrations indicate the various taxonomic orders and are credited as follows (<https://creativecommons.org/licenses/by/3.0/>): Pholidota/Certartiodactyla (Steven Traver), Carnivora (Gabriela Palomo-Munoz), Chiroptera (Margot Michaud), Lagomorpha/Marsupialia/Monotremata (Sarah Werning), Perissodactyla (Oscar Sanisidro), Primates (T. Michael Keesey), Proboscidea (Margot Michaud), Rodentia (Jiro Wada) sourced from PhyloPic (<http://phylopic.org/>).


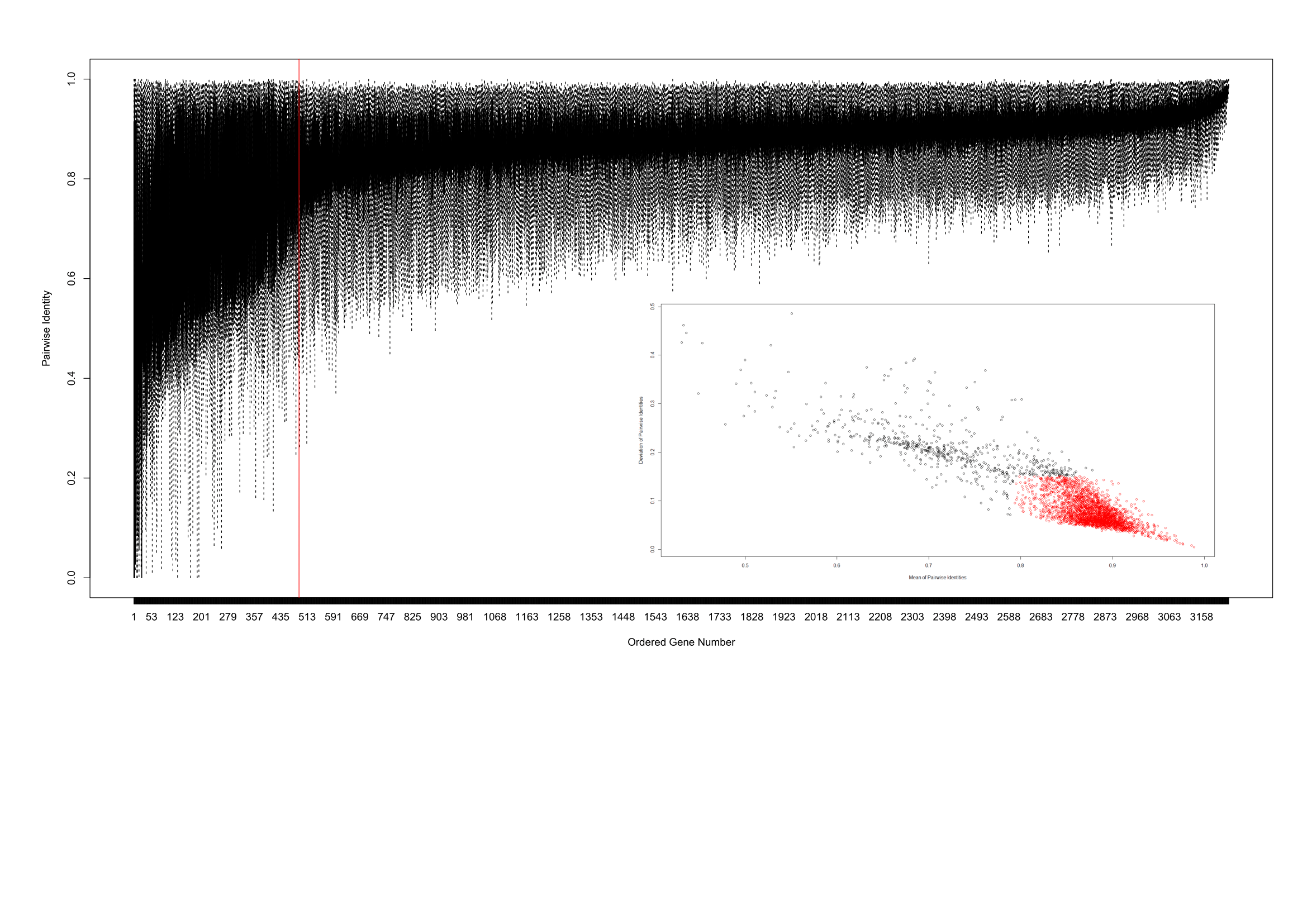


**Figure S7:** Diversity of orthologous whole-gene markers. Boxplot of whole-genes ranked from lowest (most diverse) to highest (least diverse) mean pairwise identity and the deviation of these markers (inner scatter plot). The average pairwise identity was calculated from the multiple sequence alignments of the eight pangolin species (using a single representative of each species only), by obtaining the pairwise identity for each combination of pairs and averaging it. This indicates the level of similarity (1=100% mean pairwise identity/similarity) which can be interpreted as pairwise diversity on the inverse (1=0% diverse). The vertical red line on the boxplot indicates the point (marker 566) at which genes to the left are likely outliers (values >2 Qn deviations from the median of the mean pairwise identities) as they are too diverse to be reliable (possible influence of parology, repetitive regions, bad alignment, bad gene annotation, etc.). This cutoff, along with another (values >2 Qn deviations from the median of the standard deviations of mean pairwise identities) can be viewed in the scatter plot whereby black points are the outliers (first 610 markers with the lowest mean pairwise identity or highest standard deviation of mean pairwise identity) and red points are likely reliable markers. These outlier markers have been highlighted in red in the sheet “removed genes” in the diversity database (Database S1: available at <https://doi.org/10.5281/zenodo.7517409>). The remaining points make up 3 410 610 polymorphisms from 2 623 orthologous whole-genes.

**Table S1:** Samples sequenced and references used in this study. BUSCO v5 scores were obtained from mammalian orthologues by uploading the assemblies to gVolante (<https://gvolante.riken.jp/>). Unpublished genome assemblies and sequencing data for DNA Zoo versions of *Manis pentadactyla, M. javanica,* and *Phataginus tricuspis* are used with permission from the DNA Zoo Consortium ([dnazoo.org](https://urldefense.proofpoint.com/v2/url?u=http-3A__dnazoo.org&d=DwMFaQ&c=ZQs-KZ8oxEw0p81sqgiaRA&r=Y9GzIzoqhGT1NM1qk9BvD2DV6FJmLqygwZGkyG6lk-o&m=d_CCoUKdhJ1TLeIz_vA5cdU-szZPTbdQ5woRfrubeaU&s=MJpQzTEGb9pQGca7wjpCUhzniRHkETDk-K4mfV9b2bE&e=)). These DNA Zoo draft assemblies were created and reviewed following the Hi-C method (Dudchenko, et al. 2017; Dudchenko, et al. 2018).

| **Individual** | **Genbank accession number** | **Isolate; origin** | **Study** | **Collection year; sample type; collector** | **Sequencing platform** | **BUSCO scores (completed_S; completed_D; Fragmented; Missing)** | **Estimated coverage. For samples in this study: (1) using Lander/Waterman equation with 2.45 Gb genome length as standard / (2) mapped to *P. tricuspis* DNA Zoo reference.** | **Notes** |
| --- | --- | --- | --- | --- | --- | --- | --- | --- |
| Chinese pangolin (*Manis pentadactyla*) | GCA_000738955.1 | MPE899; Taiwan | (Choo, et al. 2016) | N/A | Illumina HiSeq | 70.5; 0.7; 10; 18.8 | 59x | Unpublished DNA Zoo version with additional Hi-C and NovaSeq data can be found here: <https://www.dnazoo.org/assemblies/Manis_pentadactyla> |
| Chinese pangolin (*Manis pentadactyla*) | GCA_014570555.1 | MP20; Confiscated in Yunnan, China | (Hu, Hao, et al. 2020) | N/A | Illumina HiSeq and 10X genomic | 93.1; 1.3; 1.2; 4.4 | 281.6x | Used 77.19 Gb of the 180.66 Gb available data for mapping |
| Sunda pangolin (*Manis javanica*) | GCA_001685135.1 | MP_PG03-UM; Malaysia | (Choo, et al. 2016) | N/A | Illumina HiSeq | 74.6; 0.6; 6; 8.8 | 145.7x | Used 71.33 Gb of the 169.4 Gb available data for mapping  Unpublished DNA Zoo version with additional Hi-C and HiSeq data can be found here: <https://www.dnazoo.org/assemblies/Manis_javanica> |
| Sunda pangolin (*Manis javanica*) | GCA_014570535.1 | MJ74; Confiscated in Yunnan, China | (Hu, Hao, et al. 2020) | N/A | Illumina HiSeq and 10X genomic | 92.3; 0.9; 2; 4.8 | 411.8x | Used 112.07 Gb of the 316.2 Gb available data for mapping |
| Philippine pangolin (*Manis culionensis*) |  | MCUP0005; Casuyan, Palawan Isl., Philippines | This study | 1945; museum skin; H.H. Hoogstraal / Field Museum of Natural History, Chicago (FMNH 62919) | Illumina HiSeq X Ten | 6.2; 0; 12.5; 81.3 | (1) 76.7x / (2) 19.9x |  |
| Indian pangolin (3) (*Manis crassicaudata*) |  | MCR3; Confiscated but died in captivity, Sri Lanka | This study | 2008; tissue from dead individual; Jayanthi Alahakoon / Colombo Zoo | Illumina HiSeq X Ten | 40.9; 0.2; 16.9; 42 | (1) 53.1x / (2) 33.2x |  |
| Indian pangolin (4) (*Manis crassicaudata*) |  | MCR4; Confiscated but died in captivity, Sri Lanka | This study | 2008; tissue from dead individual; Jayanthi Alahakoon / Colombo Zoo | Illumina HiSeq X Ten | N/A | (1) 16.2x / (2) 9.1x | Only used in Heterozygosity and PSMC analyses due to limited coverage |
| *Manis sp.* | GCA_016801295.1 | Confiscated in Sichuan, China | (Cao, et al. 2021) | N/A | Illumina HiSeq | 54.5; 0.3; 13.9; 31.3 | 44x | Indicated at *M. crassicaudata* on NCBI |
| Black-bellied pangolin (*Phataginus tetradactyla*) |  | CAM085; Yaoundé bushmeat market, Cameroon | This study | 2007; tissue from dead individual; F. Njiokou & P. Gaubert | Illumina HiSeq 3000 | 55.9; 0.4; 15.3; 28.4 | (1) 43.4x / (2) 30.1x |  |
| White-bellied pangolin (*Phataginus tricuspis*) | GCA_004765945.1 | BS60 | Unpublished - BROAD institute | O. Ryder / San Diego Zoo Institute for Conservation Research | Illumina HiSeq | 65.5; 0.8; 12.4; 21.3 | 30.2x |  |
| White-bellied pangolin (*Phataginus tricuspis*) |  | Jaziri | Unpublished - DNA Zoo | Pittsburgh Zoo & PPG Aquarium | Illumina NovaSeq and Hi-C | 87.4; 1.5; 4.2; 6.9 | Unknown | The assembly can be found here courtesy of DNA Zoo: <https://www.dnazoo.org/assemblies/Phataginus_tricuspis> |
| Temminck’s pangolin (*Smutsia temminckii*) |  | STEM 81; Kalahari Oryx Game Farm, South Africa | This study | 2012; tissue from dead individual; D.W. Pietersen | Illumina HiSeq X Ten | 46.4; 0.4; 17.2; 36 | (1) 43.9x / (2) 21.9x |  |
| Giant pangolin (*Smutsia gigantea*) |  | CAM011; Yaoundé bushmeat market, Cameroon | This study | 2007; tissue from dead individual; F. Njiokou & P. Gaubert | Illumina HiSeq 3000 and Oxford Nanopore | 79.8; 1.3; 7; 11.9 | (1) 100.4x / (2) 57.4x | Mapping coverage estimate is from short read data only |

**Table S2:** Ancestral painting output statistics related to the test for hybrid individuals between various sets (trios) of putative parental species. P1and P2 refer to putative parental species one and two.

| **Hybrid test trio** | **Species** | **Homozygous genotypes P1** | **Heterozygous genotypes (P1-P2)** | **Homozygous genotypes P2** | | **Proportion of heterozygous genotypes (%)** |
| --- | --- | --- | --- | --- | --- | --- |
| Trio 1 - P1 | *M. crassicaudata* | 224566 | 0 | 0 | 0 | |
| Trio 1 - Hybrid | *Manis species* | 36814 | 4492 | 181552 | 2 | |
| Trio 1 - P2 | *M. javanica* | 0 | 0 | 224566 | 0 | |
| Trio 2 - P1 | *M. culionensis* | 210997 | 0 | 0 | 0 | |
| Trio 2 - Hybrid | *Manis species* | 56583 | 3748 | 148943 | 1.8 | |
| Trio 2 - P2 | *M. javanica* | 0 | 0 | 210997 | 0 | |
| Trio 3 - P1 | *Manis species* | 222505 | 0 | 0 | 0 | |
| Trio 3 - Hybrid | *M. culionensis* | 22313 | 9281 | 190001 | 4.2 | |
| Trio 3 - P2 | *M. javanica* | 0 | 0 | 222505 | 0 | |
| Trio 4 - P1 | *M. culionensis* | 1294988 | 0 | 0 | 0 | |
| Trio 4 - Hybrid | *M. crassicaudata* | 381209 | 11247 | 878312 | 0.9 | |
| Trio 4 - P2 | *M. javanica* | 0 | 0 | 1294988 | 0 | |

**Table S3**: Output of the concordance analysis implemented in IQ-TREE along with the significance of the Chi-squared test of independence between the two discordant gene counts (gDF1_N and gDF2_N). This was used to test whether incomplete lineage sorting (ILS) may be the sole cause of discordance for a branch whereby a significant p-value (*) indicates the contrary. Branch Clade refers to the clade onto which the branch opens in the phylogenetic tree in Figure 1. The rest of the column ID’s follow that of IQ-TREE; ID: Branch ID, gCF: Gene concordance factor (=gCF_N/gN %), gCF_N: Number of trees concordant with the branch, gDF1: Gene discordance factor for NNI-1 branch (=gDF1_N/gN %), gDF1_N: Number of trees concordant with NNI-1 branch, gDF2: Gene discordance factor for NNI-2 branch (=gDF2_N/gN %), gDF2_N: Number of trees concordant with NNI-2 branch, gDFP: Gene discordance factor due to polyphyly (=gDFP_N/gN %), gDFP_N: Number of trees decisive but discordant due to polyphyly, gN: Number of trees decisive for the branch, sCF: Site concordance factor averaged over 100 quartets (=sCF_N/sN %), sCF_N: sCF in absolute number of sites, sDF1: Site discordance factor for alternative quartet 1 (=sDF1_N/sN %), sDF1_N: sDF1 in absolute number of sites, sDF2: Site discordance factor for alternative quartet 2 (=sDF2_N/sN %), sDF2_N: sDF2 in absolute number of sites, sN: Number of informative sites averaged over 100 quartet, gEF_p: p-value of the Chi-squared test of independence for genes.

| **Branch ID** | **Branch Clade** | **gCF** | **gCF_N** | **gDF1** | **gDF1_N** | **gDF2** | **gDF2_N** | **gDFP** | **gDFP_N** | **gN** | **sCF** | **sCF_N** | **sDF1** | **sDF1_N** | **sDF2** | **sDF2_N** | **sN** | **Branch-length** | **gEF_p** |
| --- | --- | --- | --- | --- | --- | --- | --- | --- | --- | --- | --- | --- | --- | --- | --- | --- | --- | --- | --- |
| 16 | *M. culionensis*/*M. javanica* Malaysia/*M. javanica* China | 91.32 | 2957 | 3.18 | 103 | 3.68 | 119 | 1.82 | 59 | 3238 | 86.62 | 77921.4 | 7.18 | 5998.11 | 6.21 | 5318.77 | 89238.28 | 2.13912 | 0.277353521 |
| 18 | *Manis* sp*./M. culionensis/M. javanica Malaysia/M. javanica* China | 97.59 | 3160 | 0.12 | 4 | 0.06 | 2 | 2.22 | 72 | 3238 | 89.69 | 247931.1 | 5.21 | 12487.84 | 5.1 | 12177.43 | 272596.4 | 3.91262 | 0.419316578 |
| 19 | *M. crassicaudata/Manis* sp*./M. culionensis/M. javanica* Malaysia/*M. javanica* China | 94.6 | 3063 | 2.5 | 81 | 1.14 | 37 | 1.76 | 57 | 3238 | 67.99 | 166950 | 16.91 | 39310.42 | 15.1 | 35605.53 | 241866 | 2.71664 | 4.98E-05* |
| 20 | *M. pentadactyla* Taiwan/*M. pentadactyla* China | 98.67 | 3195 | 0.37 | 12 | 0.37 | 12 | 0.59 | 19 | 3238 | 96.5 | 310196.3 | 1.59 | 4394.39 | 1.91 | 5194.61 | 319785.3 | 4.16025 | 1 |
| 21 | *Manis* (Asian pangolins) | 96.15 | 2373 | 1.22 | 30 | 1.22 | 30 | 1.42 | 35 | 2468 | 81.27 | 391309.6 | 8.93 | 42936.17 | 9.79 | 46976.18 | 481221.9 | 3.04534 | 1 |
| 22 | Pholidota & Carnivora | 93.71 | 1072 | 2.8 | 32 | 3.15 | 36 | 0.35 | 4 | 1144 | 91.01 | 1235125 | 4.27 | 57919.86 | 4.72 | 64050.99 | 1357096 | 2.38006 | 0.626943182 |
| 23 | African pangolins | 96.64 | 2385 | 1.01 | 25 | 1.26 | 31 | 1.09 | 27 | 2468 | 76.93 | 345520.9 | 11.82 | 53083.68 | 11.25 | 50536.63 | 449141.2 | 3.12795 | 0.409780382 |
| 24 | *Phataginus* | 96.54 | 3127 | 1.27 | 41 | 1.08 | 35 | 1.11 | 36 | 3239 | 79.05 | 248559.4 | 10.28 | 29904.15 | 10.67 | 31191.61 | 309655.2 | 3.13571 | 0.480161224 |
| 25 | *Smutsia* | 97.44 | 3155 | 0.65 | 21 | 0.8 | 26 | 1.11 | 36 | 3238 | 84.25 | 280467 | 8.01 | 24451.21 | 7.74 | 23795.52 | 328713.7 | 3.56152 | 0.479055921 |
| 17 | *M. javanica* Malaysia/*M. javanica* China | 90.15 | 2919 | 3.74 | 121 | 4.39 | 142 | 1.73 | 56 | 3238 | 88.39 | 23207.07 | 5.95 | 1502.69 | 5.66 | 1444.31 | 26154.07 | 2.02097 | 0.19449974 |

**Table S4:** Dates used for soft bound fossil calibrations on specific nodes to be used as priors for the MCMCtree analysis of divergence estimates of pangolins. These calibrations are based on both dated fossils and molecular phylogeny estimates with reasoning provided for each. The calibration of Pholidota is the most recent calibration node possible due to the scarcity of fossils for genus/species-based estimates of the group.

| **Node** | **Date** | **Fossil** | **Reference** | **Notes** |
| --- | --- | --- | --- | --- |
| Ferae | 66–87 Ma | Min  UALVP 50993 and 50994 (Oldest stem-carnivores - miacids, viverravids)  Max  Molecular estimate | (Fox, et al. 2010)  (Zhou, et al. 2011; Gaubert, et al. 2018) | Due to no upper estimates of Ferae, we used a molecular dated calibration which has been used in previous studied |
| Carnivora | 37.3–66 Ma | Min  *Daphoenus* & *Hesperocyon*  Max  UALVP 50993 and 50994 (Oldest stem-carnivores - miacids, viverravids) | (Benton, et al. 2015)  (Fox, et al. 2010) | *Tapocyon* may  be an even older caniform; (46–43 Ma). However, it is placed outside Carnivora (Wesley‐Hunt and Flynn 2005). The oldest feliforms may  be the nimravids, but this too is uncertain (Hunt 2004). |
| Pholidota | 31–45 Ma | Min  *Manidae* (oldest manidae fossil)  Max  *Euromanis krebsi* (oldest)  *Eurotamandua joresi*  *Eomanis waldi* | (Gebo and Rasmussen 1985)  (Gaudin, et al. 2009; Rose 2012) | Messel deposits (Germany). Found with *Eurotamandua joresi* which has been debated as to whether it should be included in the Pholidota or whether it predates this order but Gaudin, et al. (2009) places it under Pholidota as sister to *Eomanis* and *Euromanis* |

**Table S5:** Estimated posterior mean or median divergence estimates and 95% Highest Posterior Density (HPD) interval of each node for this study and the one conducted by Gaubert, et al. (2018). The latter study involved mitogenomes and nine nuclear genes, included more individuals and fossil calibrations within the order Carnivora, and used the program BEAST (Bouckaert, et al. 2014) to obtain time to most recent common ancestor (TMRCA) estimates. $ refers to nodes with fossil priors for this study. * refers to nodes where the 95% HPD of divergence estimates do not overlap in the two studies.

|  | This study | | Gaubert et al. (2018) | |
| --- | --- | --- | --- | --- |
| Node | **Mean** | **95% HPD** | **Median** | **95% HPD** |
| Ferae$ | 79.47 | 67.66 – 87.24 | 78.9 | 69.6 – 87.0 |
| Carnivora$ | 49.95 | 36.49 – 65.44 | 50.8 | 44.9 – 57.4 |
| Caniformia |  |  | 41.6 | 38.0 – 46.0 |
| *Mustela – Ailuropoda* |  |  | 35.4 | 30.5 – 40.9 |
| Felidae |  |  | 11.3 | 10.0 – 13.3 |
| *Acinonyx – Felis* |  |  | 7.2 | 3.3 – 10.2 |
| Pholidota$ | 41.34 | 33.54 – 45.45 | 37.9 | 31.4 – 44.6 |
| *Smutsia - Phataginus* | 20.35 | 12.79 – 28.29 | 22.9 | 18.7 – 27.2 |
| *Manis* | 22.62 | 17.07 – 28.23* | 12.9 | 10.3 – 15.6 |
| *M. crassicaudata – (Manis* sp*, M. javanica, M. culionensis)* | 16.84 | 12.21 – 22.00* | 9.1 | 6.6 – 11.4 |
| *Manis* sp*. – (M. javanica – M. culionensis)* | 7.16 | 4.73 – 10.42 |  |  |
| *M. javanica – M. culionensis* | 2.71 | 1.70 – 4.21 | 1.7 | 0.4 – 2.5 |
| *M. javanica* China *– M. javanica* Malaysia | 0.77 | 0.46 – 1.24 |  |  |
| *M. pentadactyla* China *– M. pentadactyla* Taiwan | 2.82 | 1.48 – 4.95 |  |  |
| *Smutsia* | 9.78 | 5.55 – 15.74 | 9.8 | 5.6 – 13.2 |
| *Phataginus* | 11.34 | 6.55 – 17.45 | 13.3 | 9.3 – 16.5 |
| *P. tricuspis* |  |  | 2.7 | 0.8 – 4.6 |
| *P. tricuspis* West Africa *–* Western Central Africa |  |  | 1.1 | 0.0 – 2.4 |

**Table S6:** Proportion of genome-wide heterozygosity for mammalian species ranked from least to most diverse. As depicted in Figure S6, each mammalian order is colour-coded (Pholidota = dark red), and citations are given for the studies in which the value was originally provided for each species. Heterozygosity values for pangolins (Pholidota) include those calculated in our study as well as Hu, Hao, et al. (2020), who used a different method of calculation (using VCFtools on autosomal SNPs with 50 kb non-overlapping sliding windows). This is an edited and updated version of the table created by Hu, Hao, et al. (2020).

| **Species** | **Heterozygosity (%)** | **Mammalian order** | **Sources** |
| --- | --- | --- | --- |
| Iberian lynx (*Lynx pardinus*) | 0.010 | Carnivora | (Abascal, et al. 2016) |
| Domestic cat (*Felis catus*) | 0.012 | Carnivora | (Cho, et al. 2013) |
| Baiji (*Lipotes vexillifer*) | 0.012 | Certartiodactyla | (Zhou, et al. 2013) |
| Myanmar snub-nosed monkey (*Rhinopithecus strykeri*) | 0.015 | Primates | (Zhang, et al. 2016) |
| Altai Neanderthal (*Homo sapiens*) | 0.017 | Primates | (Prüfer, et al. 2014) |
| Cheetah (*Acinonyx jubatus*) | 0.020 | Carnivora | (Dobrynin, et al. 2015) |
| Snow leopard (*Panthera uncia syn*) | 0.023 | Carnivora | (Cho, et al. 2013) |
| Yangtze river dolphin (*Lipotes vexillifer*) | 0.026 | Certartiodactyla | (Zhou, et al. 2013) |
| Eurasian lynx (*Lynx lynx*) | 0.028 | Carnivora | (Abascal, et al. 2016) |
| Siberian tiger (*Panthera tigris altaica)* | 0.030 | Carnivora | (Dobrynin, et al. 2015) |
| Domestic dog (*Canis familiaris*) | 0.032 | Carnivora | (Lindblad-Toh, et al. 2005) |
| Tasmanian devil (*Sarcophilus harrisii*) | 0.032 | Marsupialia | (Cho, et al. 2013) |
| Black snub-nosed monkey (*Rhinopithecus bieti*) | 0.033 | Primates | (Zhou, et al. 2016) |
| Island fox (*Urocyon littoralis*) - San Miguel | 0.033 | Carnivora | (Robinson, et al. 2016) |
| Bengal tiger (*Panthera tigris tigris*) | 0.040 | Carnivora | (Dobrynin, et al. 2015) |
| Golden snub-nosed monkey (*Rhinopithecus roxellana*) | 0.042 | Primates | (Zhou, et al. 2016) |
| White lion (*Panthera leo*) | 0.048 | Carnivora | (Cho, et al. 2013) |
| Amur tiger (*Panthera tigris altaica*) | 0.049 | Carnivora | (Cho, et al. 2013) |
| Island fox (*Urocyon littoralis*) - San Nicolis | 0.049 | Carnivora | (Robinson, et al. 2016) |
| Aye-Aye (*Daubentonia madagascariensis*) | 0.051 | Primates | (Perry, et al. 2011) |
| Wild horse (*Equus ferus przewalskii*) | 0.052 | Perissodactyla | (Huang, et al. 2014) |
| African lion (*Panthera leo*) | 0.058 | Carnivora | (Cho, et al. 2013) |
| Grey snub-nosed monkey (*Rhinopithecus brelichi*) | 0.062 | Primates | (Zhou, et al. 2016) |
| Eastern lowland gorilla (*Gorilla beringei graueri*) | 0.064 | Primates | (Xue, et al. 2015) |
| Bornean orangutan (*Pongo pygmaeus*) | 0.065 | Primates | (Locke, et al. 2011) |
| Mountain gorilla (*Gorilla beringei beringei*) | 0.065 | Primates | (Xue, et al. 2015) |
| Naked mole rat (*Heterocephalus glaber*) | 0.068 | Rodentia | (Kim, et al. 2011) |
| Pileated gibbon (*Hylobates pileatus*) | 0.073 | Primates | (Carbone, et al. 2014) |
| White tiger (*Panthera tigris tigris*) | 0.073 | Carnivora | (Cho, et al. 2013) |
| White-bellied pangolin (*Phataginus tricuspis*) - DG | 0.074 | Pholidota | Sequencing data from DNAZoo |
| Dromedary (*Camelus dromedarius*) | 0.074 | Certartiodactyla | (Wu, et al. 2014) |
| Platypus (*Ornithorhynchus anatinus*) | 0.075 | Monotremata | (Warren, et al. 2008) |

| Indian pangolin (*Manis crassicaudata*) – MCR4 | 0.075 | Pholidota | This study |
| --- | --- | --- | --- |

| Eastern lowland gorilla (*Gorilla beringei graueri*) | 0.076 | Primates | (Scally, et al. 2012) |
| --- | --- | --- | --- |
| Human Han (*Homo* species) | 0.077 | Primates | (Meyer, et al. 2012) |
| West African chimpanzees (*Pan troglodytes verus*) | 0.080 | Primates | (Mikkelsen, et al. 2005) |
| African green monkey (*Chlorocebus aethiops aethiops*) | 0.080 | Primates | (Warren, et al. 2015) |
| Wild bactrian camel (*Camelus bactrianus ferus*) | 0.084 | Certartiodactyla | (Wang, et al. 2012) |
| Chinese pangolin (*Manis pentadactyla*) - Taiwan | 0.085 | Pholidota | Sequencing data from Choo, et al. (2016). Analysis from this study |
| Sunda pangolin (*Manis javanica*) - Average over 74 individuals | 0.085 | Pholidota | Samples in Hu, Hao, et al. (2020). Analysis in their study |
| Minke whale (*Balaenoptera acutorostrata*) | 0.086 | Certartiodactyla | (Yim, et al. 2014) |
| Finless porpoise (*Neophocaena phocaenoides*) | 0.086 | Certartiodactyla | (Yim, et al. 2014) |
| Koala (*Phascolarctos cinereus*) | 0.087 | Marsupialia | (Johnson, et al. 2018) |
| Tibetan antelope (*Pantholops hodgsonii*) | 0.088 | Certartiodactyla | (Ge, et al. 2013) |
| Yak (*Bos grunniens*) | 0.089 | Certartiodactyla | (Qiu, et al. 2012) |
| Mongolian horse (*Equus ferus caballus*) | 0.089 | Perissodactyla | (Huang, et al. 2014) |
| Domestic bactrian camel (*Camelus bactrianus*) | 0.090 | Certartiodactyla | (Wang, et al. 2012) |
| Southern white rhinoceros (*Ceratotherium simum simum*) | 0.090 | Perissodactyla | (Tunstall, et al. 2018) |
| Indian pangolin (*Manis crassicaudata*) – MCR3 | 0.094 | Pholidota | This study |
| Common chimpanzee (*Pan troglodytes*) | 0.095 | Primates | (Mikkelsen, et al. 2005) |
| Domestic horse (*Equus ferus caballus*) | 0.095 | Perissodactyla | (Wade, et al. 2009) |
| Cross River gorilla (*Gorilla gorilla diehli*) | 0.096 | Primates | (Xue, et al. 2015) |
| Black-bellied pangolin (*Phataginus tetradactyla*) | 0.100 | Pholidota | This study |
| Wrangel woolly mammoth (*Mammuthus primigenius*) | 0.100 | Proboscidea | (Palkopoulou, et al. 2015) |
| Polar bear (*Ursus maritimus*) | 0.108 | Carnivora | (Liu, et al. 2014) |
| Chinese pangolin (*Manis pentadactyla*) – Yunnan, China confiscation | 0.109 | Pholidota | Sequencing data from Hu, Hao, et al. (2020). Analysis from this study |
| Island fox (*Urocyon littoralis*)- San Clemente | 0.110 | Carnivora | (Robinson, et al. 2016) |
| Northern white rhinoceros (*Ceratotherium simum cottoni*) | 0.110 | Perissodactyla | (Tunstall, et al. 2018) |
| Chinese pangolin (*Manis pentadactyla*) | 0.114 | Pholidota | Sample MP20 in Hu, Hao, et al. (2020). Analysis in their study |
| Bactrian camel (*Camelus bactrianus*) | 0.116 | Certartiodactyla | (Wu, et al. 2014) |
| Sumatran orangutan (*Pongo abelii*) | 0.120 | Primates | (Xue, et al. 2015) |
| Gray fox (*Urocyon cinereoargenteus*) | 0.120 | Carnivora | (Robinson, et al. 2016) |
| Brown hyena (*Parahyaena brunnea*) | 0.121 | Carnivora | (Westbury, et al. 2018) |
| Cow (*Bos taurus*) | 0.121 | Certartiodactyla | (Corbett-Detig, et al. 2015) |
| Rat (*Rattus norvegicus*) | 0.125 | Rodentia | (Leffler, et al. 2012) |
| Oimyakon woolly mammoth (*Mammuthus primigenius*) | 0.125 | Proboscidea | (Palkopoulou, et al. 2015) |
| Chinese pangolin (*Manis pentadactyla*) - Average over 23 individuals | 0.127 | Pholidota | Samples in Hu, Hao, et al. (2020). Analysis in their study |
| Sumatran rhinoceros (*Dicerorhinus sumatrensis*) | 0.130 | Perissodactyla | (Mays, et al. 2018) |
| Giant panda (*Ailuropoda melanoleuca*) | 0.132 | Carnivora | (Li, et al. 2010) |
| Siamang (*Symphalangus syndactylus*) | 0.140 | Primates | (Carbone, et al. 2014) |
| Bottlenose dolphin (*Tursiops truncatus*) | 0.142 | Certartiodactyla | (Yim, et al. 2014) |
| Western lowland gorilla (*Gorilla gorilla gorilla*) | 0.144 | Primates | (Xue, et al. 2015) |
| Giant pangolin (*Smutsia gigantea*) | 0.146 | Pholidota | This study |
| Gray wolf (*Canis lupus*) | 0.149 | Carnivora | (Corbett-Detig, et al. 2015) |
| Fin whale (*Balaenoptera physalus*) | 0.151 | Certartiodactyla | (Yim, et al. 2014) |
| Sunda pangolin (*Manis javanica*) | 0.152 | Pholidota | Sample MJ74 in Hu, Hao, et al. (2020). Analysis in their study |
| Temminck's pangolin (*Smutsia temminckii*) | 0.155 | Pholidota | This study |
| Chinese hamster (*Cricetulus griseus*) | 0.159 | Rodentia | (Lewis, et al. 2013) |
| Asian pangolin sp. (Manis sp.) - Sichuan, China confiscated | 0.161 | Pholidota | Sequencing data from Cao, et al. (2021). Analysis in this study. |
| Silvery gibbon (*Hylobates moloch*) | 0.17 | Primates | (Carbone, et al. 2014) |
| Malaysian cynomolgus macaque (*Macaca fascicularis*) | 0.171 | Primates | (Higashino, et al. 2012) |
| Central African chimpanzees (*Pan troglodytes troglodytes*) | 0.176 | Primates | (Mikkelsen, et al. 2005) |
| Western lowland gorilla (*Gorilla gorilla gorilla*) | 0.178 | Primates | (Scally, et al. 2012) |
| Vervet monkey (*Chlorocebus aethiops pygerythrus*) | 0.180 | Primates | (Warren, et al. 2015) |
| Wild boar (*Sus scrofa*) - Tibetan | 0.182 | Certartiodactyla | (Li, et al. 2013) |
| Olive baboon (*Papio anubis*) | 0.189 | Primates | (Corbett-Detig, et al. 2015) |
| Island fox (*Urocyon littoralis*) - San Rosa | 0.191 | Carnivora | (Robinson, et al. 2016) |
| Island fox (*Urocyon littoralis*) - San Catalina | 0.196 | Carnivora | (Robinson, et al. 2016) |
| Island fox (*Urocyon littoralis*) - San Cruz | 0.197 | Carnivora | (Robinson, et al. 2016) |
| Northern white-cheeked gibbon (*Nomascus leucogenys*) | 0.220 | Primates | (Carbone, et al. 2014) |
| Bighorn sheep (*Ovis canadensis*) | 0.222 | Certartiodactyla | (Corbett-Detig, et al. 2015) |
| Sunda pangolin (*Manis javanica*) - Malaysia | 0.224 | Pholidota | Sequencing data from Choo, et al. (2016). Analysis from this study |
| Sunda pangolin (*Manis javanica*) - Yunnan, China confiscation | 0.230 | Pholidota | Sequencing data from sample MJ74 in Hu, Hao, et al. (2020). Analysis from this study |
| Alpaca (*Vicugna pacos*) | 0.266 | Certartiodactyla | (Wu, et al. 2014) |
| David’s myotis (*Myotis davidii*) | 0.279 | Chiroptera | (Zhang, et al. 2013) |
| Rhesus macaque (*Macaca mulatta*) | 0.287 | Primates | (Corbett-Detig, et al. 2015) |
| Cape buffalo (*Syncerus caffer caffer*) | 0.288 | Certartiodactyla | (de Jager, et al. 2021) |
| Brown bear (*Ursus arctos*) | 0.320 | Carnivora | (Liu, et al. 2014) |
| White-bellied pangolin (*Phataginus tricuspis*) - CWA | 0.334 | Pholidota | Sequencing data from Genbank (GCA_004765945.1). Analysis from this study |
| Common marmoset (*Callithrix jacchus*) | 0.341 | Primates | (Consortium 2014) |
| Przewalski's horse (*Equus ferus przewalskii*) | 0.363 | Perissodactyla | (Corbett-Detig, et al. 2015) |
| Brandt’s bat (*Myotis brandtii*) | 0.371 | Chiroptera | (Seim, et al. 2013) |
| Chinese rhesus macaque (*Macaca mulatta lasiota*) | 0.410 | Primates | (Yan, et al. 2011) |
| Wild boar (*Sus scrofa*) | 0.441 | Certartiodactyla | (Corbett-Detig, et al. 2015) |
| Black flying fox (*Pteropus alecto*) | 0.453 | Chiroptera | (Zhang, et al. 2013) |
| Opossum (*Monodelphis domestica*) | 0.490 | Marsupialia | (Mikkelsen, et al. 2007) |
| Philippine pangolin (*Manis culionensis*) | 0.492 | Pholidota | This study |
| Crab-eating macaque (*Macaca fascicularis*) | 0.530 | Primates | (Yan, et al. 2011) |
| Rabbit (*Oryctolagus cuniculus*) | 0.750 | Lagomorpha | (Carneiro, et al. 2014) |
| Eastern hoolock gibbon (*Hoolock leuconedys*) | 0.800 | Primates | (Carbone, et al. 2014) |
| House mouse (*Mus musculus castaneus*) | 0.809 | Rodentia | (Corbett-Detig, et al. 2015) |

**Table S7:** Estimated generation times of each species of pangolins based on the available literature. These times include a sum of the gestation period (passing on genomic information to the next generation at conception) and time until sexual maturity (when genomic information can be passed on to the next generation again). These estimates were used in the PSMC (pairwise sequentially Markovian coalescent) model analysis on pangolins in order to get the timing of changes in IICR (inverse instantaneous coalescence rate) as accurate as possible. We defined generation time as from the conception of an individual to the time until the first born of that individual is conceived.

| **Species** | **Estimated generation time** | **Evidence** | **Citations** |
| --- | --- | --- | --- |
| Chinese pangolin (*Manis pentadactyla*) | 2 years | Reaches sexual maturity at 1–1.5 years old, but may be as a low as 6–7 months; gestation is 6–7 months (captive data) | (Chin, et al. 2012; Zhang, et al. 2016) |
| Sunda pangolin (*Manis javanica*) | 1.5 years | 1 year (supposed sexual maturity of males is 1.5 years based off sperm analysis – A. Kurniawan, unpublished) until sexual maturity but may be as low as 6–7 months; gestation is 6 months (captive and seizure data) | (Zhang, et al. 2015; Zhang, et al. 2017) |
| Philippine pangolin (*Manis culionensis*) | 1.5 years | No data, but likely similar to the Sunda pangolin. |  |
| Indian pangolin (*Manis crassicaudata*) | 3 years | Up to 3 years until sexual maturity (unpublished data), gestation is around 6–8 months (135–251 days; captive data). | (Mohapatra, et al. 2018; Mahmood, et al. 2020) |
| Black-bellied pangolin (*Phataginus tetradactyla*) | 2.5 years | 2 years (unpublished suggestion – book chapter), gestation is 5 months (150 days; wild data). | (Pagès 1972a; Gudehus, et al. 2020) |
| White-bellied pangolin (*Phataginus tricuspis*) | 2.5 years | No data for maturity (probably similar to Black-bellied pangolin), gestation is 5 months (140–150 days; wild data). | (Pagès 1972b) |
| Temminck’s pangolin (*Smutsia temminckii*) | 2.5 years | Sexual maturity is likely around 2 years but no home range is established for a few more years (assuming home range is important, then 3–7 years), gestation is 3.5–4.5 months (105–140 days; both captive and wild data). | (van Ee 1966; Pietersen, et al. 2020) |
| Giant pangolin (*Smutsia gigantea*) | 3 years | No data available, but likely similar, if not longer than, Temminck’s pangolin due to larger size. |  |

**References:**

Abascal F, Corvelo A, Cruz F, Villanueva-Cañas JL, Vlasova A, Marcet-Houben M, Martínez-Cruz B, Cheng JY, Prieto P, Quesada V, et al. 2016. Extreme genomic erosion after recurrent demographic bottlenecks in the highly endangered Iberian lynx. Genome biology 17:251.

Benton MJ, Donoghue PC, Asher RJ, Friedman M, Near TJ, Vinther J. 2015. Constraints on the timescale of animal evolutionary history. Palaeontol Electron 18:1-106.

Bouckaert R, Heled J, Kühnert D, Vaughan T, Wu C-H, Xie D, Suchard MA, Rambaut A, Drummond AJ. 2014. BEAST 2: A software platform for bayesian evolutionary analysis. PLOS Computational Biology 10:e1003537.

Cao P, Dai Q, Deng C, Zhao X, Qin S, Yang J, Ju R, Wang Z, Lu G, Gu X, et al. 2021. Genome-wide signatures of mammalian skin covering evolution. Science China Life Sciences 64:1765-1780.

Carbone L, Alan Harris R, Gnerre S, Veeramah KR, Lorente-Galdos B, Huddleston J, Meyer TJ, Herrero J, Roos C, Aken B, et al. 2014. Gibbon genome and the fast karyotype evolution of small apes. Nature 513:195-201.

Carneiro M, Rubin C-J, Di Palma F, Albert FW, Alföldi J, Martinez Barrio A, Pielberg G, Rafati N, Sayyab S, Turner-Maier J, et al. 2014. Rabbit genome analysis reveals a polygenic basis for phenotypic change during domestication. Science 345:1074-1079.

Chin SC, Lien CY, Chan YT, Chen CL, Yang YC, Yeh LS. 2012. Monitoring the gestation period of rescued Formosan pangolin (Manis pentadactyla pentadactyla) with progesterone radioimmunoassay. Zoo Biology 31:479-489.

Cho YS, Hu L, Hou H, Lee H, Xu J, Kwon S, Oh S, Kim H-M, Jho S, Kim S, et al. 2013. The tiger genome and comparative analysis with lion and snow leopard genomes. Nature Communications 4:2433.

Choo SW, Rayko M, Tan TK, Hari R, Komissarov A, Wee WY, Yurchenko AA, Kliver S, Tamazian G, Antunes A, et al. 2016. Pangolin genomes and the evolution of mammalian scales and immunity. Genome Research 26:1312-1322.

Consortium TMGSaA. 2014. The common marmoset genome provides insight into primate biology and evolution. Nature Genetics 46:850.

Corbett-Detig RB, Hartl DL, Sackton TB. 2015. Natural selection constrains neutral diversity across a wide range of species. PLoS biology 13:e1002112.

de Jager D, Glanzmann B, Möller M, Hoal E, van Helden P, Harper C, Bloomer P. 2021. High diversity, inbreeding and a dynamic Pleistocene demographic history revealed by African buffalo genomes. Scientific Reports 11:4540.

Dobrynin P, Liu S, Tamazian G, Xiong Z, Yurchenko AA, Krasheninnikova K, Kliver S, Schmidt-Küntzel A, Koepfli K-P, Johnson W, et al. 2015. Genomic legacy of the African cheetah, Acinonyx jubatus. Genome biology 16:277-277.

Dudchenko O, Batra SS, Omer AD, Nyquist SK, Hoeger M, Durand NC, Shamim MS, Machol I, Lander ES, Aiden AP, et al. 2017. De novo assembly of the *Aedes aegypti* genome using Hi-C yields chromosome-length scaffolds. Science 356:92-95.

Dudchenko O, Shamim MS, Batra SS, Durand NC, Musial NT, Mostofa R, Pham M, Glenn St Hilaire B, Yao W, Stamenova E, et al. 2018. The Juicebox Assembly Tools module facilitates *de novo* assembly of mammalian genomes with chromosome-length scaffolds for under $1000. bioRxiv:254797.

Fox RC, Scott CS, Rankin BD. 2010. New early carnivoran specimens from the Puercan (Earliest Paleocene) of Saskatchewan, Canada. Journal of Paleontology 84:1035-1039.

Gaubert P, Antunes A, Meng H, Miao L, Peigné S, Justy F, Njiokou F, Dufour S, Danquah E, Alahakoon J, et al. 2018. The complete phylogeny of pangolins: Scaling up resources for the molecular tracing of the most trafficked mammals on earth. Journal of Heredity 109:347-359.

Gaudin TJ, Emry RJ, Wible JR. 2009. The phylogeny of living and extinct pangolins (mammalia, pholidota) and associated taxa: A morphology based analysis. Journal of Mammalian Evolution 16:235-305.

Ge R-L, Cai Q, Shen Y-Y, San A, Ma L, Zhang Y, Yi X, Chen Y, Yang L, Huang Y, et al. 2013. Draft genome sequence of the Tibetan antelope. Nature Communications 4:1858.

Gebo DL, Rasmussen DT. 1985. The earliest fossil pangolin (Pholidota: Manidae) from Africa. Journal of Mammalogy 66:538-541.

Gudehus M, Pietersen DW, Hoffmann M, Cassidy R, Cassidy T, Sodeinde O, Lapuente J, Assovi BG-M, Shirley MH. 2020. Black-bellied pangolin *Phataginus tetradactyla* (Linnaeus, 1766). In: Challender DWS, Nash HC, Waterman C, editors. Pangolins: Science, Society and Conservation: Academic Press. p. 123-138.

Higashino A, Sakate R, Kameoka Y, Takahashi I, Hirata M, Tanuma R, Masui T, Yasutomi Y, Osada N. 2012. Whole-genome sequencing and analysis of the Malaysian cynomolgus macaque (Macaca fascicularis) genome. Genome biology 13:R58.

Hu J-Y, Hao Z-Q, Frantz L, Wu S-F, Chen W, Jiang Y-F, Wu H, Kuang W-M, Li H, Zhang Y-P, et al. 2020. Genomic consequences of population decline in critically endangered pangolins and their demographic histories. National Science Review 7:798-814.

Hu J, Roos C, Lv X, Kuang W, Yu L. 2020. Molecular genetics supports a potential fifth Asian pangolin species (Mammalia, Pholidota, <i>Manis</i>). Zoological Science 37:538-543, 536.

Huang J, Zhao Y, Shiraigol W, Li B, Bai D, Ye W, Daidiikhuu D, Yang L, Jin B, Zhao Q, et al. 2014. Analysis of horse genomes provides insight into the diversification and adaptive evolution of karyotype. Scientific Reports 4:4958.

Hunt RM. 2004. Global climate and the evolution of large mammalian carnivores during the later Cenozoic in North America. Bulletin of the American Museum of Natural History 2004:139-156.

Johnson RN, O’Meally D, Chen Z, Etherington GJ, Ho SYW, Nash WJ, Grueber CE, Cheng Y, Whittington CM, Dennison S, et al. 2018. Adaptation and conservation insights from the koala genome. Nature Genetics 50:1102-1111.

Kim EB, Fang X, Fushan AA, Huang Z, Lobanov AV, Han L, Marino SM, Sun X, Turanov AA, Yang P, et al. 2011. Genome sequencing reveals insights into physiology and longevity of the naked mole rat. Nature 479:223-227.

Kumar S, Stecher G, Li M, Knyaz C, Tamura K. 2018. MEGA X: Molecular Evolutionary Genetics Analysis across Computing Platforms. Molecular Biology and Evolution 35:1547-1549.

Leffler EM, Bullaughey K, Matute DR, Meyer WK, Ségurel L, Venkat A, Andolfatto P, Przeworski M. 2012. Revisiting an old riddle: What determines genetic diversity levels within species? PLoS biology 10:e1001388.

Lewis NE, Liu X, Li Y, Nagarajan H, Yerganian G, O'Brien E, Bordbar A, Roth AM, Rosenbloom J, Bian C, et al. 2013. Genomic landscapes of Chinese hamster ovary cell lines as revealed by the Cricetulus griseus draft genome. Nature biotechnology 31:759-765.

Li M, Tian S, Jin L, Zhou G, Li Y, Zhang Y, Wang T, Yeung CKL, Chen L, Ma J, et al. 2013. Genomic analyses identify distinct patterns of selection in domesticated pigs and Tibetan wild boars. Nature Genetics 45:1431-1438.

Li R, Fan W, Tian G, Zhu H, He L, Cai J, Huang Q, Cai Q, Li B, Bai Y, et al. 2010. The sequence and de novo assembly of the giant panda genome. Nature 463:311-317.

Lindblad-Toh K, Wade CM, Mikkelsen TS, Karlsson EK, Jaffe DB, Kamal M, Clamp M, Chang JL, Kulbokas EJ, Zody MC. 2005. Genome sequence, comparative analysis and haplotype structure of the domestic dog. Nature 438:803-819.

Liu S, Lorenzen Eline D, Fumagalli M, Li B, Harris K, Xiong Z, Zhou L, Korneliussen Thorfinn S, Somel M, Babbitt C, et al. 2014. Population Genomics Reveal Recent Speciation and Rapid Evolutionary Adaptation in Polar Bears. Cell 157:785-794.

Locke DP, Hillier LW, Warren WC, Worley KC, Nazareth LV, Muzny DM, Yang S-P, Wang Z, Chinwalla AT, Minx P, et al. 2011. Comparative and demographic analysis of orang-utan genomes. Nature 469:529-533.

Mahmood T, Mohapatra RK, Perera P, Irshad N, Akrim F, Andleeb S, Waseem M, Sharma S, Panda S. 2020. Indian pangolin Manis crassicaudata (Geoffroy, 1803). In: Challender DWS, Nash HC, Waterman C, editors. Pangolins: Science, Society and Conservation: Academic Press. p. 71-88.

Mays HL, Hung C-M, Shaner P-J, Denvir J, Justice M, Yang S-F, Roth TL, Oehler DA, Fan J, Rekulapally S, et al. 2018. Genomic Analysis of Demographic History and Ecological Niche Modeling in the Endangered Sumatran Rhinoceros Dicerorhinus sumatrensis. Current Biology 28:70-76.e74.

Meyer M, Kircher M, Gansauge M-T, Li H, Racimo F, Mallick S, Schraiber JG, Jay F, Prüfer K, de Filippo C, et al. 2012. A high-coverage genome sequence from an archaic Denisovan individual. Science 338:222-226.

Mikkelsen T, Hillier L, Eichler E, Zody M, Jaffe D, Yang S-P, Enard W, Hellmann I, Lindblad-Toh K, Altheide T. 2005. Initial sequence of the chimpanzee genome and comparison with the human genome. Nature 437:69-87.

Mikkelsen TS, Wakefield MJ, Aken B, Amemiya CT, Chang JL, Duke S, Garber M, Gentles AJ, Goodstadt L, Heger A, et al. 2007. Genome of the marsupial Monodelphis domestica reveals innovation in non-coding sequences. Nature 447:167-177.

Mohapatra R, Panda S, Sahu S. 2018. On the gestation period of Indian pangolins (Manis crassicaudata) in captivity. Biodiversity Int J 2:559-560.

Pagès E. 1972a. Comportement agressif et sexuel chez les pangolins arboricoles (Manis tricuspis et M. longicauda). Biol. Gabonica 1:3-62.

Pagès E. 1972b. Comportement maternal et developpement du j'eune chez un pangolin ar-boricole (M. tricuspis). Biol. Gabon 8:63-120.

Palkopoulou E, Mallick S, Skoglund P, Enk J, Rohland N, Li H, Omrak A, Vartanyan S, Poinar H, Götherström A, et al. 2015. Complete genomes reveal signatures of demographic and genetic declines in the Woolly Mammoth. Current Biology 25:1395-1400.

Perry GH, Reeves D, Melsted P, Ratan A, Miller W, Michelini K, Louis EE, Jr, Pritchard JK, Mason CE, Gilad Y. 2011. A genome sequence resource for the Aye-Aye (Daubentonia madagascariensis), a nocturnal lemur from Madagascar. Genome biology and evolution 4:126-135.

Pietersen DW, Jansen R, Swart J, Panaino W, Kotze A, Rankin P, Nebe B. 2020. Temminck’s pangolin Smutsia temminckii (Smuts, 1832). In: Challender DWS, Nash HC, Waterman C, editors. Pangolins: Science, Society and Conservation: Academic Press. p. 175-193.

Prüfer K, Racimo F, Patterson N, Jay F, Sankararaman S, Sawyer S, Heinze A, Renaud G, Sudmant PH, De Filippo C. 2014. The complete genome sequence of a Neanderthal from the Altai Mountains. Nature 505:43-49.

Qiu Q, Zhang G, Ma T, Qian W, Wang J, Ye Z, Cao C, Hu Q, Kim J, Larkin DM, et al. 2012. The yak genome and adaptation to life at high altitude. Nature Genetics 44:946-949.

Robinson Jacqueline A, Ortega-Del Vecchyo D, Fan Z, Kim Bernard Y, vonHoldt Bridgett M, Marsden Clare D, Lohmueller Kirk E, Wayne Robert K. 2016. Genomic flatlining in the endangered Island Fox. Current Biology 26:1183-1189.

Rose KD. 2012. The importance of Messel for interpreting Eocene Holarctic mammalian faunas. Palaeobiodiversity and Palaeoenvironments 92:631-647.

Scally A, Dutheil JY, Hillier LW, Jordan GE, Goodhead I, Herrero J, Hobolth A, Lappalainen T, Mailund T, Marques-Bonet T, et al. 2012. Insights into hominid evolution from the gorilla genome sequence. Nature 483:169-175.

Seim I, Fang X, Xiong Z, Lobanov AV, Huang Z, Ma S, Feng Y, Turanov AA, Zhu Y, Lenz TL, et al. 2013. Genome analysis reveals insights into physiology and longevity of the Brandt’s bat Myotis brandtii. Nature Communications 4:2212.

Tunstall T, Kock R, Vahala J, Diekhans M, Fiddes I, Armstrong J, Paten B, Ryder OA, Steiner CC. 2018. Evaluating recovery potential of the northern white rhinoceros from cryopreserved somatic cells. Genome Research 28:780-788.

van Ee CA. 1966. A note on breeding the Cape pangolin Manis temniincki at Bloemfontein Zoo. International Zoo Yearbook 6:163-164.

Wade C, Giulotto E, Sigurdsson S, Zoli M, Gnerre S, Imsland F, Lear T, Adelson D, Bailey E, Bellone R. 2009. Genome sequence, comparative analysis, and population genetics of the domestic horse. Science 326:865-867.

Wang Z, Ding G, Chen G, Sun Y, Sun Z, Zhang H, Wang L, Hasi S, Zhang Y, Li J, et al. 2012. Genome sequences of wild and domestic bactrian camels. Nature Communications 3:1202-1202.

Warren WC, Hillier LW, Graves JAM, Birney E, Ponting CP, Grützner F, Belov K, Miller W, Clarke L, Chinwalla AT. 2008. Genome analysis of the platypus reveals unique signatures of evolution. Nature 453:175.

Warren WC, Jasinska AJ, García-Pérez R, Svardal H, Tomlinson C, Rocchi M, Archidiacono N, Capozzi O, Minx P, Montague MJ. 2015. The genome of the vervet (Chlorocebus aethiops sabaeus). Genome Research 25:1921-1933.

Wesley‐Hunt GD, Flynn JJ. 2005. Phylogeny of the carnivora: Basal relationships among the carnivoramorphans, and assessment of the position of ‘miacoidea’ relative to carnivora. Journal of Systematic Palaeontology 3:1-28.

Westbury MV, Hartmann S, Barlow A, Wiesel I, Leo V, Welch R, Parker DM, Sicks F, Ludwig A, Dalén L, et al. 2018. Extended and continuous decline in effective population size results in low genomic diversity in the world's rarest hyena species, the brown hyena. Molecular Biology and Evolution 35:1225-1237.

Wu H, Guang X, Al-Fageeh MB, Cao J, Pan S, Zhou H, Zhang L, Abutarboush MH, Xing Y, Xie Z, et al. 2014. Camelid genomes reveal evolution and adaptation to desert environments. Nature Communications 5:5188.

Xue Y, Prado-Martinez J, Sudmant PH, Narasimhan V, Ayub Q, Szpak M, Frandsen P, Chen Y, Yngvadottir B, Cooper DN. 2015. Mountain gorilla genomes reveal the impact of long-term population decline and inbreeding. Science 348:242-245.

Yan G, Zhang G, Fang X, Zhang Y, Li C, Ling F, Cooper DN, Li Q, Li Y, van Gool AJ, et al. 2011. Genome sequencing and comparison of two nonhuman primate animal models, the cynomolgus and Chinese rhesus macaques. Nature biotechnology 29:1019-1023.

Yim H-S, Cho YS, Guang X, Kang SG, Jeong J-Y, Cha S-S, Oh H-M, Lee J-H, Yang EC, Kwon KK, et al. 2014. Minke whale genome and aquatic adaptation in cetaceans. Nature Genetics 46:88-92.

Zhang F, Wu S, Yang L, Zhang L, Sun R, Li S. 2015. Reproductive parameters of the sunda pangolin Manis javanica. Folia Zoologica 64:129-135.

Zhang F, Wu S, Zou C, Wang Q, Li S, Sun R. 2016. A note on captive breeding and reproductive parameters of the Chinese pangolin, Manis pentadactyla Linnaeus, 1758. ZooKeys 2016:129-144.

Zhang F, Yu J, Wu S, Li S, Zou C, Wang Q, Sun R. 2017. Keeping and breeding the rescued Sunda pangolins (*Manis javanica*) in captivity. Zoo Biology 36:387-396.

Zhang G, Cowled C, Shi Z, Huang Z, Bishop-Lilly KA, Fang X, Wynne JW, Xiong Z, Baker ML, Zhao W. 2013. Comparative analysis of bat genomes provides insight into the evolution of flight and immunity. Science 339:456-460.

Zhou X, Meng X, Liu Z, Chang J, Wang B, Li M, Wengel PO-t, Tian S, Wen C, Wang Z. 2016. Population genomics reveals low genetic diversity and adaptation to hypoxia in snub-nosed monkeys. Molecular Biology and Evolution 33:2670-2681.

Zhou X, Sun F, Xu S, Fan G, Zhu K, Liu X, Chen Y, Shi C, Yang Y, Huang Z, et al. 2013. Baiji genomes reveal low genetic variability and new insights into secondary aquatic adaptations. Nature Communications 4:2708.

Zhou X, Xu S, Xu J, Chen B, Zhou K, Yang G. 2011. Phylogenomic analysis resolves the interordinal relationships and rapid diversification of the laurasiatherian mammals. Systematic Biology 61:150-150.
